# Development of Self‐Aligned Top‐Gate Transistor Arrays on Wafer‐Scale Two‐Dimensional Semiconductor

**DOI:** 10.1002/advs.202415250

**Published:** 2025-02-20

**Authors:** Yuxuan Zhu, Jinshu Zhang, Hui Xie, Yin Xia, Xiangqi Dong, Saifei Gou, Zhejia Zhang, Xinliu He, Haojie Chen, Mingrui Ao, Qicheng Sun, Yan Hu, Yuchen Tian, Jieya Shang, Yufei Song, Jiahao Wang, Sen Wang, Xiaofei Yue, Chunxiao Cong, Lihui Zhou, Sheng Dai, Zihan Xu, Jing Wan, Haibing Qiu, Yin Wang, Xiaojun Tan, Wenzhong Bao

**Affiliations:** ^1^ School of Microelectronics Fudan University Shanghai 200433 P. R. China; ^2^ School of Information Science and Technology Fudan University Shanghai 200433 P. R. China; ^3^ Key Laboratory for Advanced Materials and Feringa Nobel Prize Scientist Joint Research Center Institute of Fine Chemicals School of Chemistry & Molecular Engineering East China University of Science and Technology Shanghai 200237 P. R. China; ^4^ Shenzhen Six carbon Technology Shenzhen 518055 P. R. China; ^5^ School of Semiconductor and Physics North University of China Taiyuan 030051 P. R. China; ^6^ Shaoxin Laboratory Shaoxing 312000 P. R. China

**Keywords:** 2D semiconductors, optimization process, self‐aligned, top‐gate, wafer‐scale

## Abstract

Two‐dimensional semiconductor materials (2DSM) effectively mitigate the short‐channel effect due to their atomic thickness, offering significant advantages over traditional silicon‐based materials, particularly in short channel length. In manufacturing 2DSM top‐gate field‐effect transistors (TG‐FETs), simultaneous miniaturization of the gate and channel can only be achieved through a self‐alignment process, enabling high‐density integration of short‐channel FETs. However, current self‐aligned FETs based on 2DSM face challenges in attaining wafer‐scale integration due to manufacturing process limitations. This work has successfully developed high‐performance and wafer‐scale TG‐FET arrays using a self‐aligned method that integrates the processes of dry etching, wet selective etching, and post‐device optimization. The miniaturization is demonstrated by fabricating TG‐FETs with a channel length of 200 nm, achieving an impressive on‐state current density of 465.5 µA µm^−1^ and a high on‐off current ratio of 10^8^. Furthermore, we constructed the inverters and logic modules based on self‐aligned FETs, showcasing the process's compatibility for future integration.

## Graphical abstract

The fabrication of a wafer‐scale self‐aligned MoS_2_ FETs array is presented, with gate and channel lengths scaling simultaneously. High‐performance devices have been achieved through the optimization of contact and dielectric. Logic modules and devices with 200 nm channel length demonstrate the process's feasibility and scalability, which provides a pathway for 2D materials in miniaturization and integration.

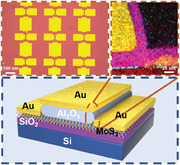

## 1 Introduction

Two‐dimensional semiconductor materials (2DSM), particularly transition metal dichalcogenides (TMDs) like MoS_2_ and WSe_2_, are pivotal in advancing integrated circuits (ICs) to the next technology node. This is largely due to their unique properties, including the absence of dangling bonds,^[^ ^1^ ^]^ ultrathin thickness,^[^ ^2^ ^]^ and excellent compatibility with silicon.^[^ ^3^ ^]^ These features help effectively mitigate the short‐channel effect, allowing device mobility to remain intact even as miniaturization progresses.^[^ ^4^ ^]^ To date, high‐quality wafer‐scale TMD films have already been successfully synthesized by chemical vapor deposition (CVD)^[^ ^5^ ^]^ and metal–organic chemical vapor deposition techniques.^[^ ^6^ ^]^ Application of TMDs have been demonstrated in logic,^[^ ^7^ ^]^ analog,^[^ ^8^ ^]^ sensing,^[^ ^9^ ^]^ memory,^[^ ^10^ ^]^ and heterostructure units,^[^ ^11^ ^]^ showcasing their extensive potentials. However, current applications of TMDs are mainly limited to channel lengths of approximately 10 µm or longer and larger gate regions. Therefore, it is essential to explore fabrication processes supporting scaling down the 2DSM top‐gate field‐effect transistors (TG‐FETs) while ensuring compatibility with large‐scale integration methods.

The integration processes for 2DSM FETs are categorized into two primary approaches^[^ ^4c^ ^]^: the gate‐first and the gate‐last processes. In the gate‐first process, the buried gate^[^ ^12^ ^]^ or global back‐gate^[^ ^13^ ^]^ is prepared before other components, while the gate‐last process involves fabricating the source‐drain and dielectric layers before the gate‐aligning deposition.^[^ ^14^ ^]^ However, both approaches encounter limitations regarding large‐scale fabrication, particularly miniaturizing channel lengths. The gate‐first process necessitates the transfer of large‐scale MoS_2_ after the gate and dielectric layers deposition, which can lead to yield loss due to potential cracks and residues formed during this additional step.^[^ ^5a^ ^]^ Conversely, the gate‐last process presents challenges for establishing the oxide seeding layer and dielectric layer because of the MoS_2_^’^s dangling‐bond‐free surface and the unavoidable steps formed between the channel and the source‐drain metal. This increased complexity makes achieving precise alignment between the gate and source‐drain regions progressively more difficult as channel lengths decrease.^[^ ^15^ ^]^ Significant overlap or underlap of the gate and source‐drain regions might increase parasitic capacitance or resistance,^[^ ^8^, ^16^ ^]^ limiting the current integration processes' ability to support further miniaturization of channel lengths.

From the inception of the silicon industry up to the 45 nm node, the polysilicon self‐aligned process emerged as a replacement for the aluminum (Al) based‐gate structure. This advancement facilitated the alignment of the gate and source‐drain regions through direct ion implantation, significantly reducing parasitic capacitance.^[^ ^17^ ^]^ Following this, self‐aligned techniques such as the lightly doped drain process and self‐aligned silicide (SALICIDE) technologies were introduced.^[^ ^18^ ^]^ The fabrication of 2DSM FETs through the self‐alignment process allows further miniaturization of the channel by overcoming the dielectric and transfer challenges while preserving the aforementioned advantages. However, traditional silicon‐based techniques, including ion implantation and selective dry etching, have limitations when applied to the inherently thin atomic thickness of the 2DSM, complicating their use in producing self‐aligned TG‐FETs with 2DSM.^[^ ^19^ ^]^ Recently, several novel self‐aligned processes targeting 2DSM FETs have emerged. One promising approach involves using nanowires as a TG hard mask to facilitate the self‐aligned deposition of the source‐drain metal, with the nanowires' width defining the channel length.^[^ ^20^ ^]^ Yet, the inconsistent arrangement and size of nanowire growth pose significant challenges in meeting the requirements for large‐scale integration. Additionally, self‐aligned FETs have been developed using a hard mask formed by the self‐oxidation of the Al top gate.^[^ ^21^ ^]^ However, this self‐oxidation process is sensitive to temperature and environmental factors, which can compromise device uniformity. Moreover, the oxide‐coated Al used as a charge‐trapping layer often leads to significant hysteresis effects. Despite the successful production of self‐aligned TG‐FETs of 2DSM with excellent performance using alternative methods like exfoliation and transfer,^[^ ^16^, ^22^ ^]^ these processes face significant challenges regarding compatibility and scalability.

In this work, we proposed a novel self‐aligned process for TG‐FETs based on monolayer CVD‐grown MoS_2_. The innovative method utilizes the TG metal as a hard mask and implements a wet/dry hybrid etching technique on the dielectric layer to achieve source‐drain metal self‐aligned deposition. This approach showcases excellent compatibility and promotes large‐scale integrated fabrication. Post‐device optimization through annealing and encapsulation was implemented to reduce contact resistance and mitigate defects, enhancing the electrical performance and stability of the devices. Comparative analysis of both as‐fabricated and optimized self‐aligned MoS_2_ TG‐FETs array demonstrates a significant improvement in the on‐state current, I_on_/I_off_ ratio, and reductions in the subthreshold swing (*SS*) and hysteresis window. With a channel length of 200 nm, self‐aligned MoS_2_ TG‐FETs have been prepared by further scaling down the gate length (L_g_), achieving a maximum output current of 465.6 µA µm^−1^ and an impressive *I*_on_/*I*_off_ ratio of 10^8^. Additionally, inverters, NAND, and NOR logic gates have been fabricated from self‐aligned MoS_2_ TG‐FETs, highlighting the potential for further miniaturization and promising applications in advanced electronic circuits.

## 2 Results and Discussion

### 2.1 Fabrication and Characteristics of the Self‐Aligned Top‐Gate MoS_2_ Devices

**Figure 1:**
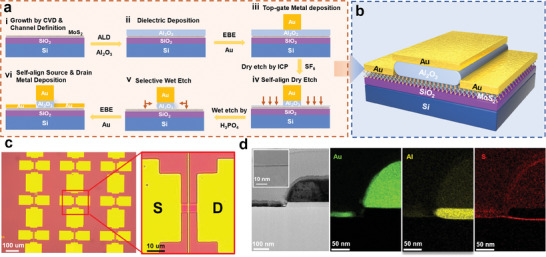
Fabrication and structure of self‐aligned MoS_2_ TG‐FETs. a) Schematic illustration of the fabrication process of self‐aligned MoS_2_ TG‐FETs. b) Schematic structure of the self‐aligned MoS_2_ TG‐FETs. c) Optical images of self‐aligned MoS_2_ TG‐FETs array. d) STEM and EDS images of contact and gate region of the self‐aligned MoS_2_ TG‐FETs.

**Figure** **1**a illustrates a schematic representation of the fabrication process for a single self‐aligned MoS_2_ TG‐FET. For further details of the array fabrication process and optical images based on 2‐inch MoS_2_ wafer, refer to Figure S1 (Supporting Information), which displays additional testing pad deposition and etch isolation steps. To establish a large‐scale device fabrication process, we first created a uniform wafer‐scale monolayer of MoS_2_ on the surface of SiO_2_/Si (300 nm thermally grown oxide) substrates by CVD. Figure S2a (Supporting Information) illustrates the photograph of the monolayer MoS_2_ grown on the 2‐inch SiO_2_/Si wafer and the selection of uniformly characterization regions. Figure S2b (Supporting Information) shows the spatial intensity mapping of the A_1g_ Raman mode, highlighting a microscopic scale uniformity. The histograms and Gaussian fit of Raman and Photoluminescence (PL) characterization displayed in Figure S2c–f (Supporting Information) of 25 selected positions reveal a narrow distribution, indicating the high uniformity of the MoS_2_ film. The PL peaks appear around 1.84 eV with a standard deviation of 0.04 eV, aligning with the A exciton peaks in the k‐valley of the Brillouin zone, indicating a direct bandgap of monolayer MoS_2_.^[^ ^23^ ^]^ Additionally, the Raman spectra demonstrate an average separation of E^1^_2g_–A_1g_ peaks of approximately 19 cm^−1^ with a standard deviation of 0.2 cm^−1^, which is in line with the previous results for monolayer MoS_2_.^[^ ^24^ ^]^ For the first step, we deposit the connection pad and alignment marker, enhancing the precision of the large‐scale fabrication, as shown in Figure S3 (Supporting Information). A seeding layer and 30 nm Al_2_O_3_ were deposited directly after the lithographic patterning and reactive ion etching to define the channel region, bypassing the source‐drain lithography step of the gate‐last process. The L_g_ of the self‐aligned TG‐FETs was established, ranging from 200 nm to 1 µm, utilizing a 150 nm thick Au layer as both the gate and a hard mask for etching the Al_2_O_3_ layer. The dielectric layer was thinned 27 nm via anisotropic dry self‐aligned etching of SF_6_, during which the gate metal served as a hard mask, as shown in Figure S4a (Supporting Information). A high selectivity ratio etching scheme was implemented to ensure optimal contact between the source‐drain metal and MoS_2_ and thin the dielectric layer. While H_3_PO_4_ exhibits a high etching rate for Al_2_O_3_, this process is inherently limited by the isotropic nature of wet etching.^[^ ^25^ ^]^ Thus, dry etching was utilized to thin the Al_2_O_3_ layer to minimize the wet etching duration, thereby guaranteeing the reliability of the gate dielectric for improving alignment. Figure S5 (Supporting Information) highlights the effect of varying wet etching time on the morphology of the dielectric following a 120 s dry etching with a 1 mL:10 mL H_3_PO_4_‐to‐water solution at 45 °C. The optimal wet etching time was approximately 4 min, as illustrated in Figures S4b and S5b (Supporting Information), which depict a height difference of 5 nm after 4 min of wet etching. Upon completing the etching process to expose the MoS_2_, a 10 nm Au layer was deposited as the contact metal for the source‐drain region, utilizing the gate metal as a hard mask for self‐alignment. The schematic of the self‐aligned MoS_2_ TG‐FETs is shown in Figure 1b, where the top gate completely covers the dielectric layer thinned by dry and wet etching, and the source‐drain metal establishes self‐aligned contact with MoS_2_. This self‐aligned TG‐FET structure promotes the simultaneous scaling of the gate and channel regions, thereby ensuring gate alignment accuracy and eliminating parasitic capacitance between the gate and source‐drain. Figure 1c illustrates an array of self‐aligned MoS_2_ TG‐FETs fabricated using a 1 µm channel length. Figure 1d presents the scanning transmission electron microscopy (STEM) images of the device cross‐section, with the inset confirming the monolayer MoS_2_. The energy dispersive spectrometer (EDS) displays the distribution of Au, Al, and S elements, confirming that the Au gate covers the Al_2_O_3_ layer. This self‐aligned structure facilitates high‐precision deposition of the source‐drain metal, minimizes the risk of a short‐circuit with the gate, and reduces the underlap region between the source‐drain and the gate to just 10 nm. Overall, the self‐aligned fabrication process eliminates the need for transfer and supports the fabrication of large‐scale FET arrays based on CVD‐grown MoS_2_.

### 2.2 Optimization of the Devices by Annealing and Encapsulation

**Figure 2:**
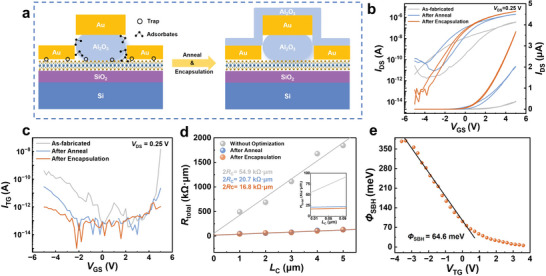
Schematic and electrical performance of the as‐fabricated and optimized self‐aligned MoS_2_ TG‐FET. a) Schematic of the self‐aligned MoS_2_ TG‐FET fabrication process. The transfer characteristics b), the gate leakage current c), and the contact resistance d) of the as‐fabricated MoS_2_ TG‐FET, the MoS_2_ TG‐FET after annealing and MoS_2_ TG‐FET after encapsulation. e) The extracted Schottky barrier height (SBH) of the optimized device.

The electrical performance of the self‐aligned MoS_2_ TG‐FETs has been comprehensively characterized. During the etching step of the gate dielectric, damage inevitably occurs, exposing the sides of Al_2_O_3_ to the environment. The lateral regions of the dielectric layer are particularly vulnerable to environmental adsorption, such as H_2_O and O_2_, which can lead to the formation of trap states.^[^ ^26^ ^]^ Moreover, residues from chemical solvents on the MoS_2_ also introduce additional trap states.^[^ ^27^ ^]^ The schematic diagram on the left side of **Figure** **2**a presents the impact of these traps and adsorbates. Figure 2b exhibits the transfer curves of the devices. During the gate voltage (*V*_TG_) scans, the carriers of the as‐fabricated device are trapped by the surface states of the impurities, resulting in a counterclockwise hysteresis window of 2.72 V extracted from the transfer curve. The contact resistance of the device is affected by the chemical solvent residue in the source‐drain region, leading to an on‐state current of only around 10^−7^ A. Additionally, defects in the dielectric of the as‐fabricated device result in a maximum gate leakage of 15 nA, as illustrated in Figure 2c. All the measurements were conducted under atmospheric conditions, with a *V*_TG_ ranging from −5 to 5 V and a drain bias (*V*_DS_) of 0.25 V. Optimization processes were implemented to improve the contact characteristics and eliminate the dielectric defects, as shown in the right side of Figure 2a. The devices underwent annealing at 200 °C for 2 h in a 5% H_2_/95% Ar atmosphere to clean the dielectric, metal, and MoS_2_ interface and remove residues (Figure 2a).^[^ ^2^ ^]^ After annealing, the devices were encapsulated with 20 nm Al_2_O_3_ via atomic layer deposition (ALD) to repair the dielectric and prevent the re‐adsorption of impurities from the environment. The electrical performances of the same device after annealing and encapsulation are exhibited in Figure 2b,c.

The transfer curves of the optimized treatment device, as shown in Figure 2b, demonstrate a significant increase in on‐state current, with values of 2.03 and 3.68 µA compared to 380 nA for the as‐fabricated device. This enhancement is primarily due to the improved contact from annealing in the H_2_/Ar atmosphere and the ALD encapsulation process, improving the contact optimization with additional high‐temperature annealing. Figure 2d evaluates the contact resistance of the device using the transfer length method, where the total resistance is fitted to the L_g_ at a gate voltage of 5 V, demonstrating a satisfactory linear fit. Extrapolation reveals that the contact resistance decreased from 27.5 to 10.4 kΩ·µm after device annealing and further to 8.4 kΩ·µm after encapsulation, which is consistent with the trend of the increased on‐state current.

The optimization process contributes to the dielectric's repair and significantly reduces hysteresis. As shown in Figure 2b, the hysteresis decreased from 2.72 to 1.50 V after annealing and decreased to 0.28 V after encapsulation. Moreover, the gate leakage reduced from 15 nA to 1.1 pA after optimization (Figure 2c), which further indicates the effectiveness of the optimization process in improving device performance. The results indicate that annealing effectively passivates defects, while encapsulation improves the dielectric quality by preventing impurities' adsorption, thus enhancing the reliability of the devices.^[^ ^28^ ^]^ Figure S6 (Supporting Information) demonstrates the optimized device's transfer curves stored in an Ar atmosphere glovebox for 10, 20, 30, and 45 days, demonstrating that the encapsulation treatment minimizes degradation of the transfer curves, significantly improving the stability of the device. The transfer curves were measured over a temperature range of 110–250 K at a *V*_DS_ of 0.2 V to extract the Schottky barrier of the optimized device, as shown in Figure S7a (Supporting Information). The result indicates that the on‐state current decreases with decreasing temperature due to the suppression of thermally emitted electrons. The Arrhenius function is obtained by fitting the temperature‐dependent transfer curve (Figure S7b, Supporting Information). Figure 2e presents the extracted barriers at varying *V*_TG_, revealing a linear decrease in the contact barriers with increasing *V*_TG_. Notably, a deviation in the initial slope occurs when the *V*_TG_ exceeds approximately 0.5 V, indicating the critical point at the flat‐band voltage. The potential barrier is extracted as 64.6 meV, representing the Schottky barrier for this contact. This value is lower than that reported in previous studies of top contact with Au deposited by electronic beam evaporation (EBE) and MoS_2_,^[^ ^29^ ^]^ suggesting that the Al_2_O_3_ wet etching process leaves no residue and that the optimized device exhibits superior contact properties.

### 2.3 Comparison and Statistical Analysis of Devices Performance

**Figure 3:**
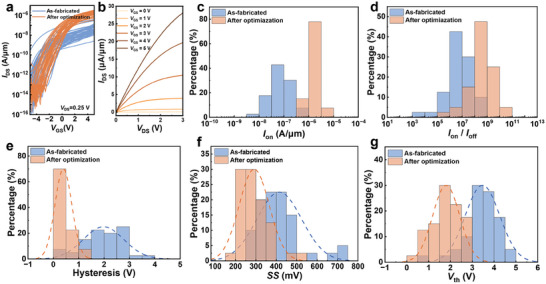
Comparison of the electrical performance of as‐fabricated and optimized self‐aligned MoS_2_ TG‐FETs. a) The transfer curves (*V*_DS_ = 0.25 V) for 40 as‐fabricated and optimized self‐aligned MoS_2_ TG‐FETs. b) The output curves of an optimized self‐aligned MoS_2_ TG‐FET. Statistical distribution of *I*_on_ c), *I*_on_/*I*_off_ d), hysteresis e), *SS* f), and *V*_th_ g) of the as‐fabricated and optimized self‐aligned MoS_2_ TG‐FETs. The mean ± standard deviation of hysteresis, *SS*, *V*_th_ are 1.98 ± 0.80 V, 414.6 ± 110.1 mV dec^−1^, and 3.47 ± 0.69 V for as‐fabricated devices, respectively. For optimized devices, the mean ± standard deviation are 0.39 ± 0.33 V, 291.7 ± 73.7 mV dec^−1^, and 1.80 ± 0.62 V, respectively.

To further evaluate the homogeneity and stability of the process optimization, we conducted comparative tests on 40 as‐fabricated and 40 optimized self‐aligned MoS_2_ TG‐FETs, each with a channel length of 1 µm. **Figure** **3**a presents the transfer curves of these FETs at *V*_DS_ = 0.25 V and *V*_TG_ ranging from −5 to 5 V. All devices exhibit typical n‐type characteristics. Notably, the optimized devices' on‐state current (*I*_on_) is more concentrated, showing an increase of 1–2 orders of magnitude compared to the as‐fabricated devices. Figure 3b illustrates the optimized device's output curves, while the typical curves of as‐fabricated and optimized devices are depicted in Figure S8 (Supporting Information). The current extracted from the output curves of the aforementioned devices increased from 3.4 to 28 µA µm^−1^ at *V*_DS_ = 3 V and *V*_TG_ = 5 V, highlighting the improvement in contact performance. We then analyzed and compared other crucial electrical parameters under identical measurement conditions (Figure 3c–g). As shown in Figure 3c, nearly 85% of the optimized devices exhibit a higher *I*_on_ than 1 µA µm^−1^, with an average value of 1.7 µA µm^−1^. In contrast, the maximum on‐state current for as‐fabricated devices is 0.48 µA µm^−1^, with a distribution ranging from nA µm^−1^ to µA µm^−1^ level. This significant disparity is primarily due to contact resistance, underscoring the effectiveness and uniformity achieved through the optimization process. The optimized devices also exhibit a rightward shift in the *I*_on_/*I*_off_ ratio distribution by approximately two orders of magnitude, with 92.5% achieving a ratio higher than 10^7^ (Figure 3d). This improvement is primarily attributed to the enhancement of the on‐state current.

Furthermore, 70% of the optimized devices exhibit hysteresis less than 0.5 V, averaging at 0.4 V, notably lower than the 2 V average hysteresis window observed in the as‐fabricated devices (Figure 3e). The average *SS*_min_ of the optimized devices decreased from 414.5 to 291.7 mV dec^−1^, as illustrated in Figure 3f. Additional dielectric thickness and type adjustments may be necessary to achieve a smaller SS. Figure 3g shows that the optimized devices' average *V*_th_ via the linear extrapolation method has decreased to 1.8 V, compared to 3.5 V for the non‐optimized devices, attributed to the reduced trap states. We also obtained *V*_th_ for both devices via the constant subthreshold current method (100 pA µm^−1^·W/L),^[^ ^30^ ^]^ with the results shown in Figure S9 (Supporting Information). The optimized devices have an average *V*_th_ of −1.49 V, while the unoptimized devices have an average of −1.81 V, consistent with the relative values obtained through the linear extrapolation method. The optimized devices exhibit a more concentrated *V*_th_ distribution, attributed to the defect density decrease. Overall, the significant optimization of these parameters demonstrates the success of the annealing and encapsulation processes in enhancing contact performance, reducing defects, improving device stability, and lowering the operating voltage, thus ensuring uniformity and applicability across the devices. To verify the universality of our approach, we have successfully fabricated self‐aligned TG‐FETs using graphene and WSe_2_ as channel materials via optimized process, shown in Figure S10 (Supporting Information). The corresponding transfer curves in Figure S10b,d (Supporting Information) exhibit graphene's high on‐current and WSe_2_’s p‐type characteristics.

### 2.4 Scaling Capability and Circuit Application of the Devices

**Figure 4:**
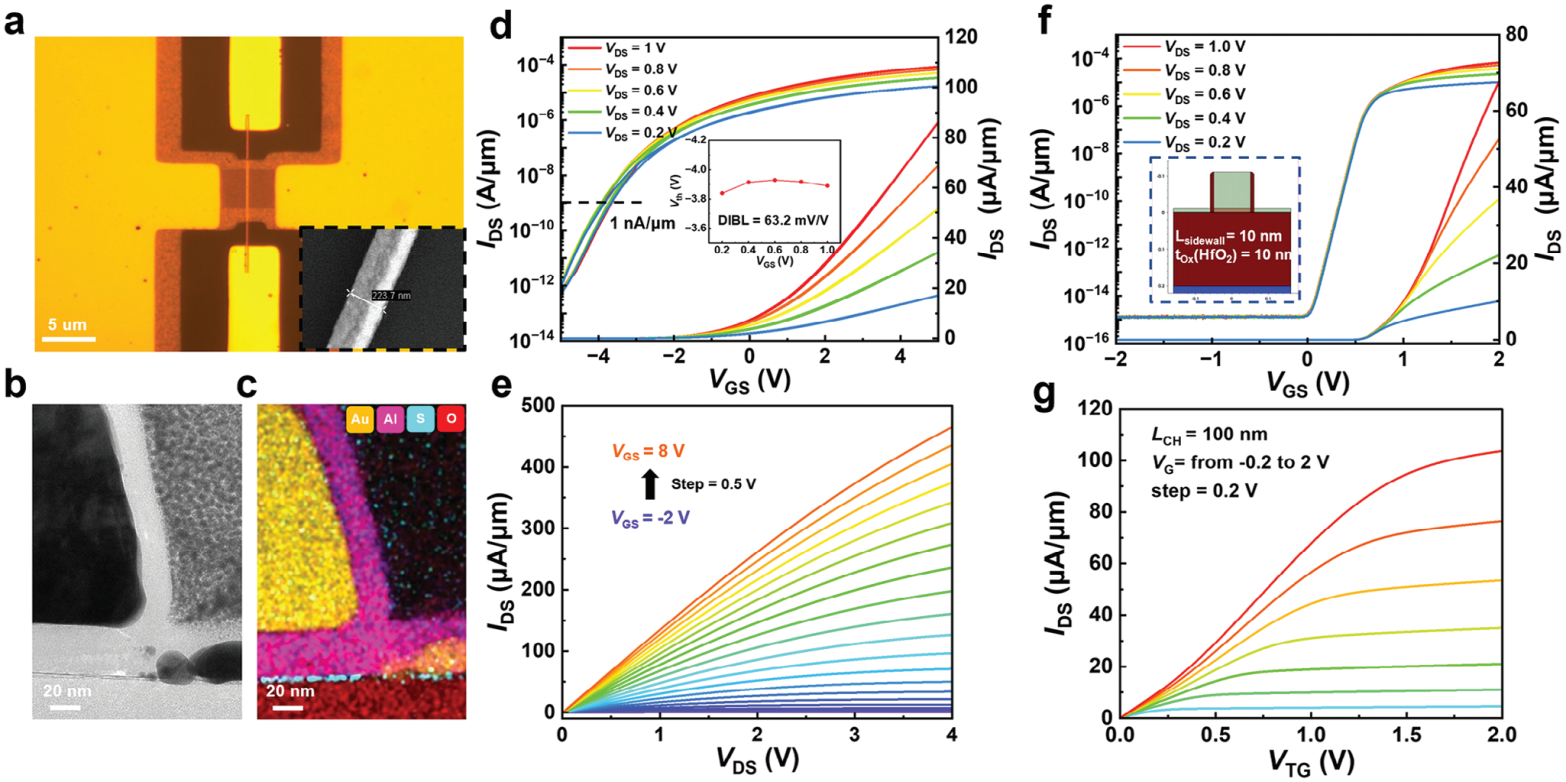
The structure and electrical performance of optimized self‐aligned MoS_2_ TG‐FETs with the submicron length of the channel. a) The optical and SEM inset images of the self‐aligned MoS_2_ TG‐FET with around 200 nm channel length. The STEM b) and EDS c) images of the contact region of optimized self‐aligned MoS_2_ TG‐FET with around 200 nm channel length. The transfer d), DIBL (d, inset), and output e) curves of the MoS_2_ TG‐FET with around 200 nm channel length. The transfer f) and output g) curves of self‐aligned MoS_2_ TG‐FET with 100 nm channel length by simulation.

We fabricated the self‐aligned top‐gate structure with submicron channel length using the optimized process to evaluate its electrical characteristics. The L_g_ directly defines the channel length of self‐aligned devices. Moreover, we employed electron beam lithography (EBL) to reduce the L_g_ and realize shorter channel self‐aligned TG‐FETs. **Figure** **4**a depicts the optical image of the device with the L_g_ defined by EBL, while the inset scanning electron microscope (SEM) image illustrates the device with a L_g_ of 223.7 nm. Figure 4b displays a STEM image of a short‐channel device, illustrating how the drain region is separated from the gate to form a self‐aligned structure. The minimal gate underlap effectively reduces the contact resistance of the ungated regions and parasitic capacitance of the overlap regions. The EDS image in Figure 4c illustrates the elemental distribution of the different layers, with a 20 nm Al_2_O_3_ encapsulation layer tightly adhering to the device surface. The device's transfer and output curves are depicted in Figure 4d,e. The device achieves a maximum on‐state current of up to 86.2 µA µm^−1^ with the highest *I*_on_/*I*_off_ ratio of over 10^8^. Moreover, negligible hysteresis in the transfer curves under the *V*_TG_ sweep are from −5 to 5 V at *V*_DS_ = 1 V, indicating that the optimized processes remain compatible with submicron channel length devices. The drain‐induced barrier lowering () is also evaluated based on *V*_th_ at different *V*_DS_ with a constant *I*_DS_ of 1 nA µm^−1^, regarded as a key parameter for short‐channel devices. The ultralow DIBL value of 63.2 mV V^−1^, shown in Figure 4e inset, along with the stable *V*_th_ and *SS* as the channel length is scaled from 800 to 200 nm in Figure S11 (Supporting Information) highlights the excellent immunity to short‐channel effects of MoS_2_ TG‐FETs. The linear output characteristics demonstrate excellent contact with the highest output current of 465.4 µA µm^−1^, underscoring a significant improvement over the device with 1 µm channel length.

To achieve scaling below 200 nm channel length while maintaining performance and yield, we will optimize the process by selecting semi‐metallic source/drain materials to reduce the contact resistance, using higher‐k dielectric and thinning the dielectric to reduce effective oxide thickness and improve the *SS*. In order to prevent gate‐source/drain short‐circuits, a low‐k dielectric layer with good step coverage should be deposited, followed by selective etching for vertical gate sidewalls. We conducted technology computer‐aided design (TCAD) simulations to show the devices of L_g_ reduces from 100 to 10 nm with the sidewall, in Figure S12a–c (Supporting Information). Figure 4f,g presents the transfer and output curves of the device with 100 nm channel length and 10 nm sidewall thickness, confirming scaling capability with simulated *SS* around 77 mV dec^−1^ and exceptional *I*_on_/*I*_off_ ratio exceeding 10^10^. Furthermore, Figure S12d,e (Supporting Information) exhibit the increase in current from 67.8 to 134.6 µA µm^−1^ (*V*_TG_ = 2 V, *V*_DS_ = 1 V) as the L_g_ reduces from 100 to 10 nm, *SS* remaining around 65 mV dec^−1^, offering insights for further optimization and scalability.

**Figure 5:**
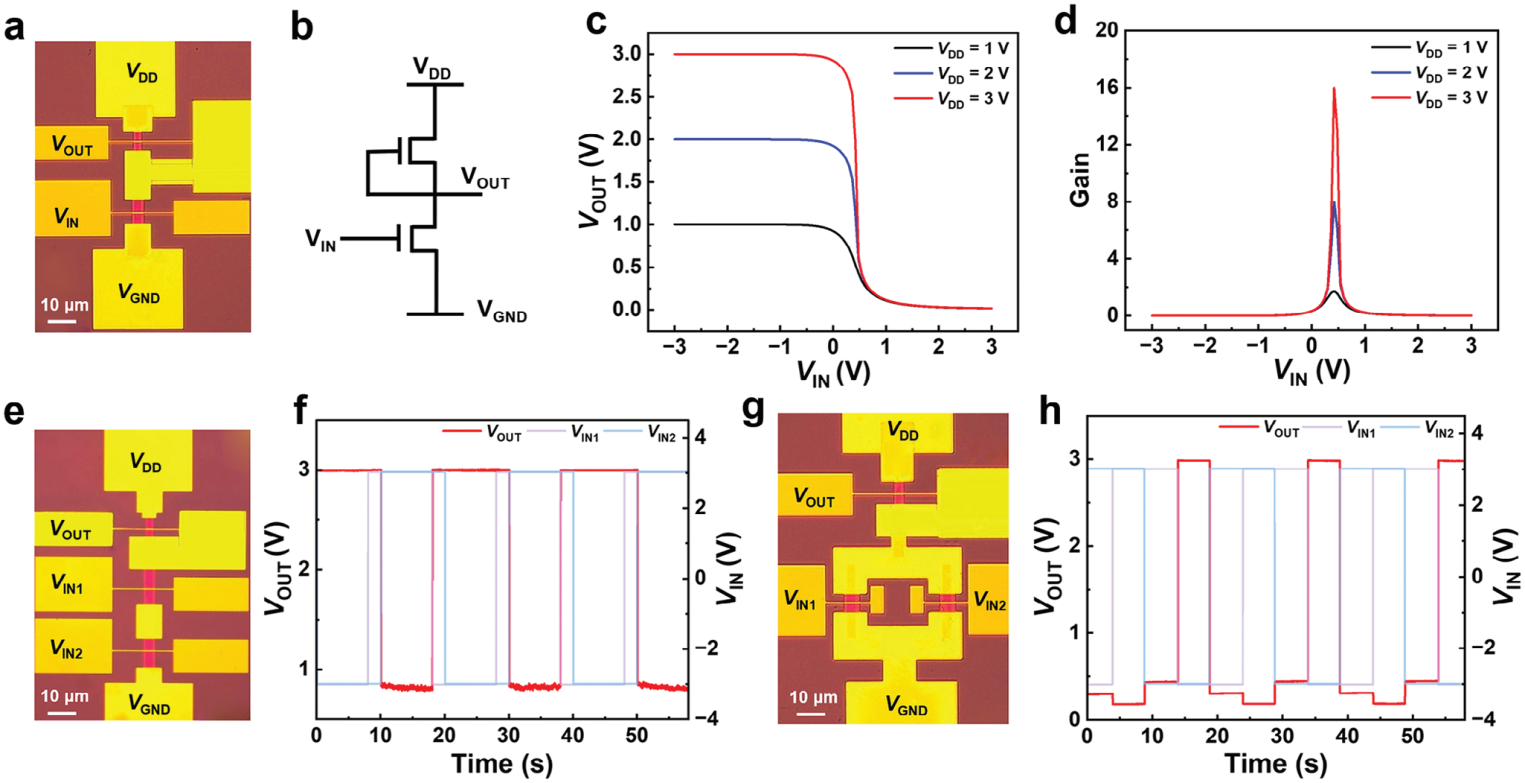
Optical images and logic functions of circuits based on the optimized self‐aligned MoS_2_ TG‐FETs. The optical image a) and the circuit diagram b) of the inverter. Voltage transfer characteristics c) and the corresponding voltage d) gains of the inverter at *V*_DD_ from 1 to 3 V. Optical images of NAND e) and NOR g) logic circuits based on the self‐aligned MoS_2_ TG‐FETs and dynamic response of NAND f) and NOR logic h), respectively.

The preceding sections have shown that the self‐aligned MoS_2_ TG‐FETs are both feasible and uniform, which is crucial for large‐scale ICs. To investigate the potential applications of this structure, we fabricated inverters based on the n‐type load and driver transistors, designed with different aspect ratios of 1 µm channel length for both transistors to investigate the potential applications of this structure. **Figure** **5**a presents the optical image and circuit diagram of the inverter. The Al_2_O_3_ layer was thoroughly etched, except for the gate dielectric of self‐aligned devices. By simply connecting the drain and gate, the load transistor can function as a nonlinear resistor without needing an additional via etching step. The detailed device fabrication and characterization methods section and Figure S13 (Supporting Information) provide the fabrication steps. Figure 5c illustrates the voltage transfer characteristics of the inverter as a function of the input voltage (*V*_IN_) under a bias voltage (*V*_DD_) ranging from 1 to 3 V. In contrast, a transition point (*V*_T_) of the high and low levels occurs at approximately 0.4 V, remaining consistent with different *V*_DD_. Figure 5d presents the resultant voltage gain (d*V*_OUT_/d*V*_IN_), exhibiting that the gain peak increases as *V*_DD_ rises, reaching a maximum of 16 at *V*_DD_ = 3 V. Moreover, Figure S14 (Supporting Information) presents the noise margin of the inverter. Since the *V*_T_ is higher than 0 V, the inverter can support multi‐level logic circuits under positive *V*_DD_. The logic units, including NAND and NOR gates, were fabricated by connecting the transistors to prove the practicability of the inverters. Figure 5e,g exhibit the corresponding optical images. Figure 5f,h also demonstrate the desired logic functions of the logic circuits from −3 to 3 V. Due to the negligible parasitic capacitance advantage of the self‐aligned structure, the NAND and NOR units display correct outputs at higher frequencies of 1 Hz and 10 Hz, as shown in Figure S15 (Supporting Information). The yields for the NAND and NOR logic devices in Figure S16 (Supporting Information) were 65% and 75%, respectively. Although achieving higher integration densities through simple width‐to‐length adjustments remains challenging, we are now focusing on optimizing doping strategies^[^ ^31^ ^]^ which are compatible with the self‐aligned process to enhance both yield and integration density.

## 3 Conclusion

This study introduced a fabrication strategy to achieve wafer‐scale self‐aligned MoS_2_ TG‐FET. This process utilizes top‐gate metal as a hard mask combined with wet and dry selective etching to achieve scaled gate and channel length while preventing overlap or underlap between the gate and source/drain. This precision is not currently possible through traditional gate‐last and gate‐first processes. The devices produced were optimized for contact and dielectric to achieve high performance and uniformity. Improvements included on‐state current, *I*_on_/*I*_off_, hysteresis window, SS, and a reduced *V*_th_. To demonstrate the effectiveness of this miniaturization approach, we fabricated devices with channel lengths around 200 nm, exhibiting on‐state currents as high as 465.6 µA µm^−1^ and an *I*_on_/*I*_off_ ratio exceeding 10^8^. Additionally, the inverters and logic units were fabricated using this technique, showcasing the process feasibility and scalability, which is crucial for advancing high‐performance 2D electronic devices in miniaturization and integration applications.

## 4 Experimental Section

### Synthesis of Wafer‐Scale MoS_2_

A monolayer MoS₂ film was synthesized via the previously reported chemical vapor deposition (CVD) process.^[^ ^32^ ^]^ In this process, the MoO₃ powder (Alfa Aesar, 99.95%) and the appropriate quantity of sulfur powder (Alfa Aesar, 99.999%) were placed in Zone 1 and Zone 2, approximately 30 cm apart, respectively. Zone 1 was placed upstream of the flow within the tube. The SiO_2_/Si substrate (heavily p‐doped Si with 300 nm SiO_2_) was positioned downstream and face‐to‐face on top of the MoO_3_ powder. Subsequently, Zone 1 and Zone 2 were heated to 650 and 180°C in an argon atmosphere as carrier gas. A continuous monolayer MoS_2_ film was synthesized at atmospheric pressure with a sulfuration time of 10 min.

### Fabrication of the MoS_2_ Self‐Aligned Top‐Gate FET

The MoS_2_ self‐aligned top‐gate FETs and circuits were based on the wafer‐scale MoS_2_ film on SiO_2_/Si substrate (Figure S1a, Supporting Information). The contact pad was patterned by laser direct writing technology (Micro‐Writer ML3) and subsequently deposited 35 nm Au using EBE (Figure S1b, Supporting Information). Then, O_2_ plasma etching was used to define the channel region through reactive ion etching (RIE; Figure S1c, Supporting Information). A seeding layer of Al_2_O_3_ was deposited by EBE and annealed in an oxygen atmosphere at 100 °C for 30 min, after which a 30 nm Al_2_O_3_ dielectric layer was grown by ALD (Figure S1d, Supporting Information). The top gate of 150 nm Au was defined by Micro‐Writer ML3 of 1 µm length or EBL of sub‐micron length and deposited by EBE (Figure S1e, Supporting Information). Then, the Al_2_O_3_ layer was thinned by 120 s SF_6_ plasma etching using inductively coupled plasma at 600 W power, 55 sccm SF_6,_ and 0.8 Pa (Figure S1f, Supporting Information). The remaining Al_2_O_3_ layer was etched using a solution of H_3_PO_4_ (10 mL H_2_O:1 mL 99.5%H_3_PO_4_) for 4 min (Figure S1g, Supporting Information) to fabricate the mask structure. Finally, the top gate was the mask to facilitate EBE's self‐aligned deposition of 10 nm Au to form the source and drain regions.

### Optimization of MoS_2_ Self‐Aligned Top‐Gate FETs

The as‐fabricated MoS_2_ self‐aligned top‐gate FETs were annealed at 5%H_2_/95%Ar atmosphere under 200 °C for 2 h. Subsequently, ALD deposited 20 nm Al_2_O_3_ for encapsulation.

### Characterization and Electrical Measurement

The PL and Raman spectroscopy of CVD‐grown MoS_2_ were obtained through a confocal Raman system (WITec Alpha300 R) with a 532 nm laser wavelength. An objective lens of 100× magnification and 0.95 numerical aperture (NA) was used. Moreover, the laser spot was ≈500 nm in diameter. The 2‐inch was divided into five small circular regions of ∼1.7 cm in diameter to demonstrate the homogeneity of the film. Subsequently, five points of 500 nm × 500 nm were selected for Raman and PL characterization, distributed uniformly at the periphery and center of the small circular regions, resulting in 25 samples.

The electrical characteristics of MoS_2_ FETs and circuits were evaluated in a probe station connected to an Agilent B1500A semiconductor analyzer and an Agilent 33 622 An arbitrary‐waveform generator in an ambient atmosphere. Temperature‐dependent properties were evaluated using a liquid nitrogen‐cooled cryogenic probe station. To confirm the device structure, a cross‐sectional high‐angle annular dark field scanning transmission electron microscopy analysis was conducted using a Titan 80–300 microscope (Thermo‐Fisher, USA) equipped with an energy‐dispersive X‐ray spectroscopy analyzer.

### Statistical Analysis

The electrical performance data of the as‐fabricated and optimized FETs for *n* devices (*n* = 40) were summarized and performed as Gauss distribution graphs without pre‐processing. The mean ± standard deviation values were calculated and marked in the corresponding captions. All data and graph processing was conducted using Origin 2024 software The *I*_on_ of the device is derived from the transfer characteristics at *V*_TG_ = 5 V. The difference quantifies the hysteresis window between the forward and reverse sweep voltages at a 1 nA µm^−1^ current from the transfer curve. The *SS* is calculated based on the following equation:

$SS=\frac{dV_{\mathrm{TG}}}{d\log_{10}I_{\mathrm{DS}}},$ with the *SS*_min_ in the linear region. The *V*_th_ is extracted via the linear extrapolation method, where V_th_ is determined by linearly extending the *I*_DS_–*V*_GS_ transfer curves at the point (*V*_0_, *I*_0_) with max transconductance (g_m_) to the *V*_GS_ axis and subtract 1/2 *V*_DS_, as: $V_{\mathrm{th}}=V_{0}\;-\frac{I_{0}}{g_{m}}-\frac{1}{2}V_{\mathrm{DS}}$.

## Conflict of Interest

The authors declare no conflict of interest.

## Supporting information



Supporting Information

## Data Availability

The data that support the findings of this study are available from the corresponding author upon reasonable request.
