# Supplementary material for: Development of Self‐Aligned Top‐Gate Transistor Arrays on Wafer‐Scale Two‐Dimensional Semiconductor
Source: Adv Sci (Weinh). 2025 Feb 20;12(15):2415250. doi: 10.1002/advs.202415250 (PMC12005750; doi:10.1002/advs.202415250)
Supplement: Supplementary file 1 — Supporting Information [file ADVS-12-2415250-s001.docx]

Supporting Information

**Development of Self-Aligned Top-Gate Transistor Arrays on Wafer-Scale Two-Dimensional Semiconductor**

*Yuxuan Zhu^†^, Jinshu Zhang^†^, Hui Xie^†^, Yin Xia, Xiangqi Dong, Saifei Gou, Zhejia Zhang, Xinliu He, Haojie Chen, Mingrui Ao, Qicheng Sun, Yan Hu, Yuchen Tian, Jieya Shang, Yufei Song, Jiahao Wang, Sen Wang, Xiaofei Yue, Chunxiao Cong, Lihui Zhou, Sheng Dai, Zihan Xu, Jing Wan,* *Haibing Qiu, Yin Wang^*^, Xiaojun Tan^*^, and Wenzhong Bao^*^*

^†^These authors contributed to this work equally.


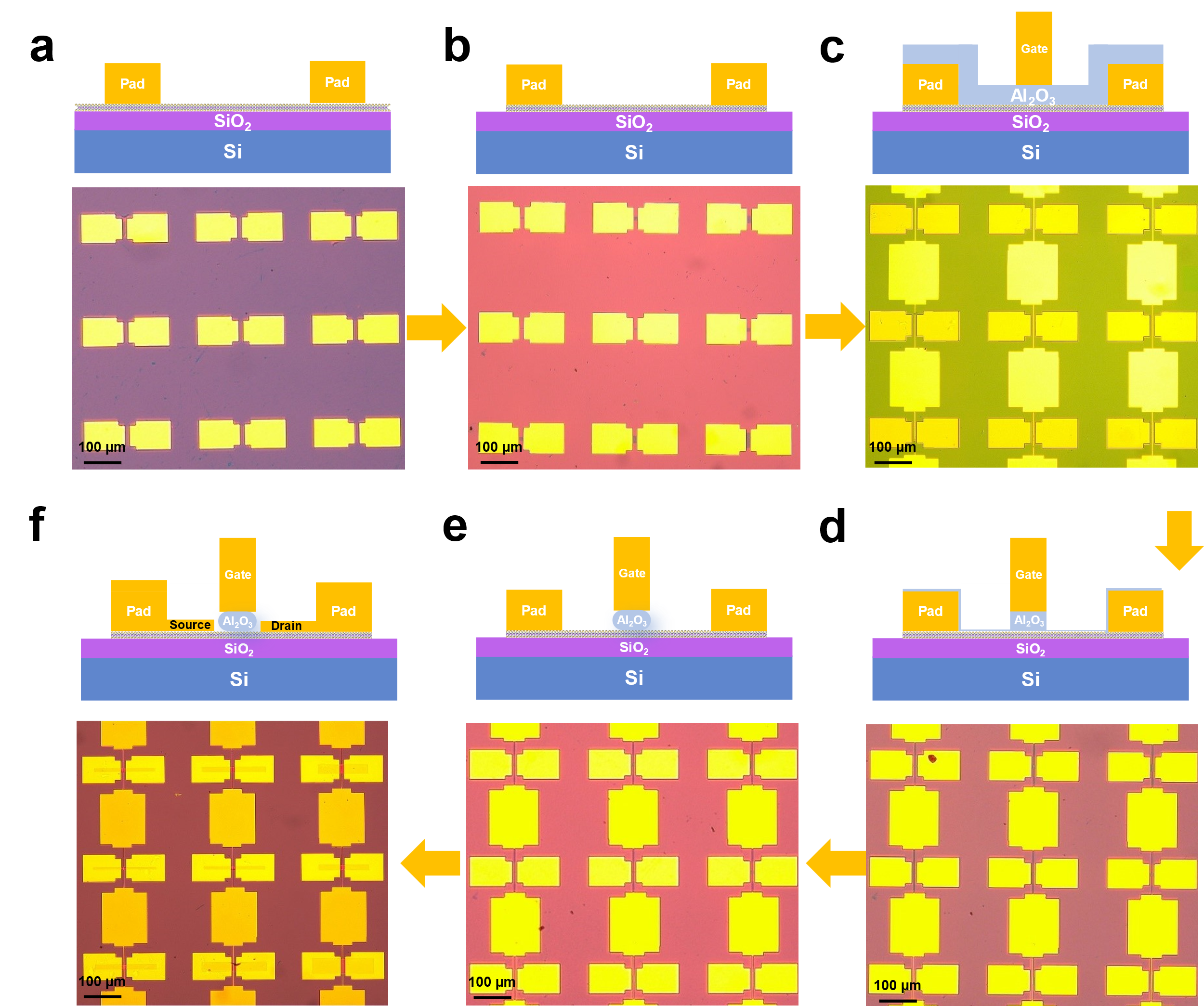


**Figure S1**. Optical images and schematic of each fabrication process of self-aligned MoS_2_ top-gate transistor array at the same scale: (a) The deposition of the pads for connection; (b) The etching of the MoS_2_ channel for isolation; (c) The deposition of the Al_2_O_3_ dielectric and the Au top gate; (d) The dry etching of the Al_2_O_3_ dielectric by SF_6_ via ICP; (e) The wet etching of the remaining Al_2_O_3_ dielectric by H_3_PO_4_; (f) The self-aligned deposition of a thin Au film for source and drain contacts.


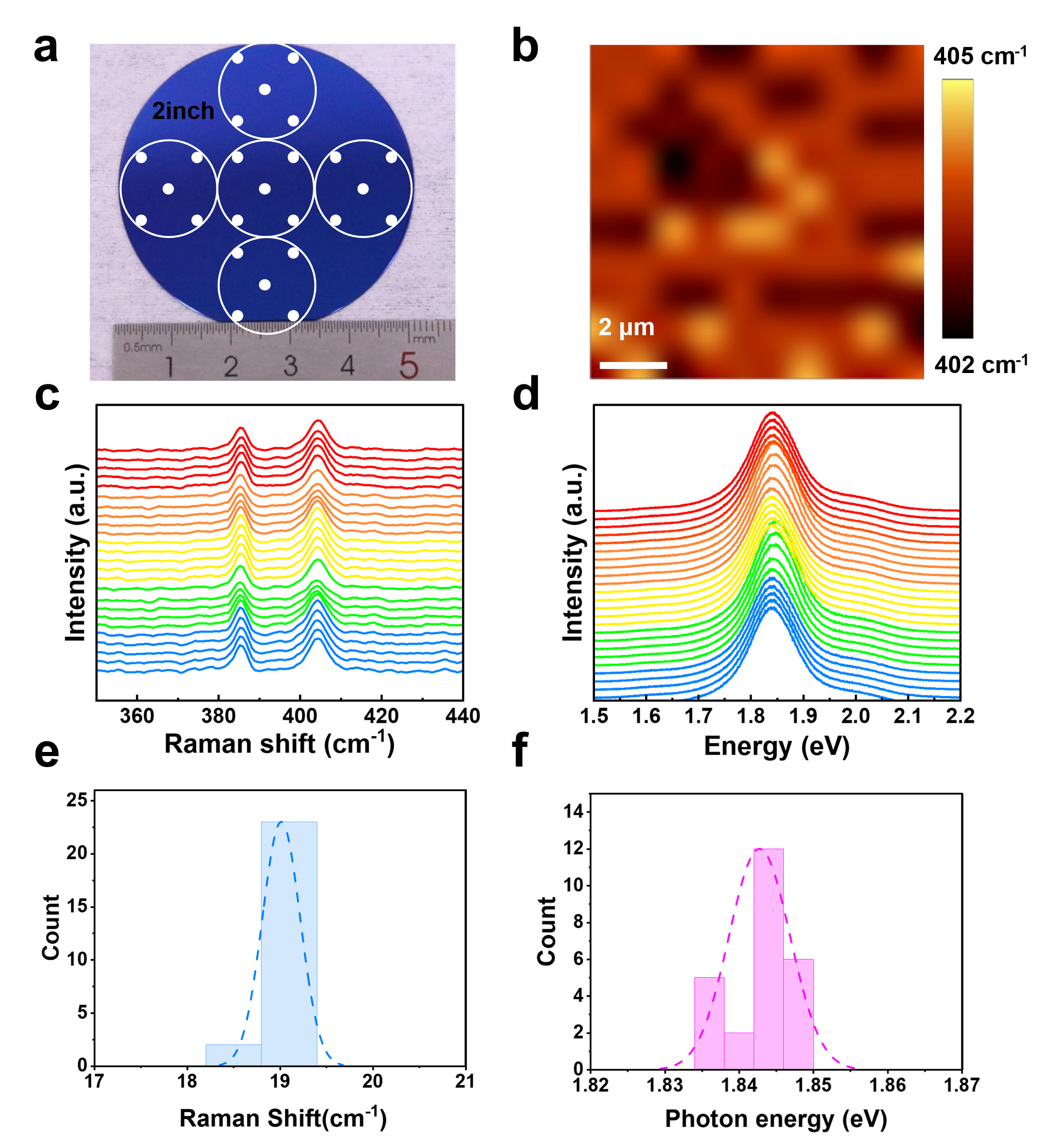


**Figure S2.** (a) Optical image and characterization regions of MoS_2_ grown on 2 inch Si/SiO_2_ substrate; (b) Spatial intensity mapping of the A_1g_ Raman mode for the center spot of the film, the color bar represents the position of A_1g_ peak; The Raman (c) and PL (d) spectra of the 25 selected regions of the CVD-grown MoS_2_. Histograms and Gaussian fits of the peak difference between the E^1^_2g_ and A_1g_ in the Raman spectra (e) and the peak position in the PL spectra (f).
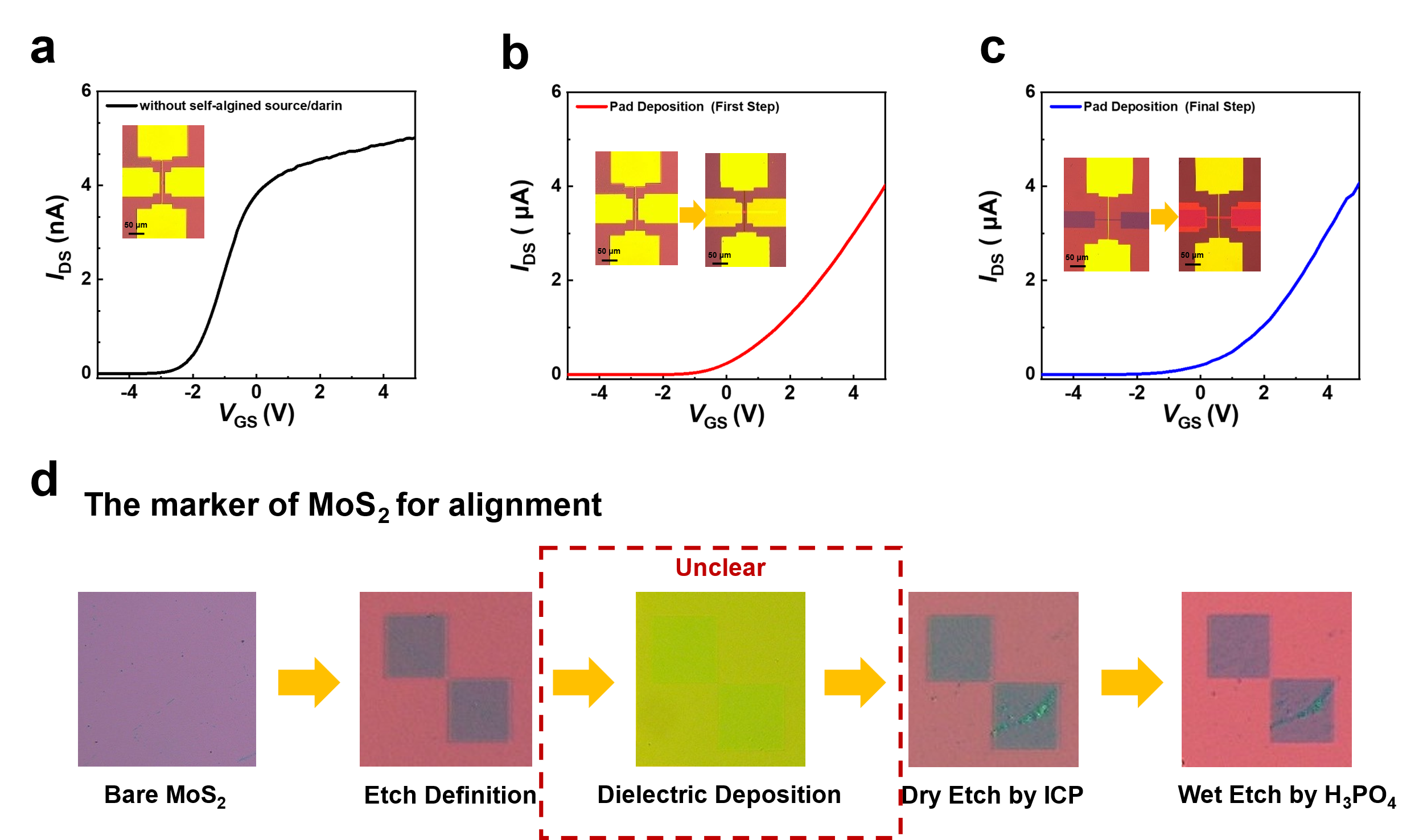


**Figure S3.** Optical images and transfer curves at *V*_DS_ = 0.25 V of the device without self-aligned source/drain contacts (a), the device with the first step of connection pad deposition (b) and the device with the final step of connection pad deposition (c); (d) Optical images of the MoS₂ film serve as a marker for alignment throughout the fabrication process.


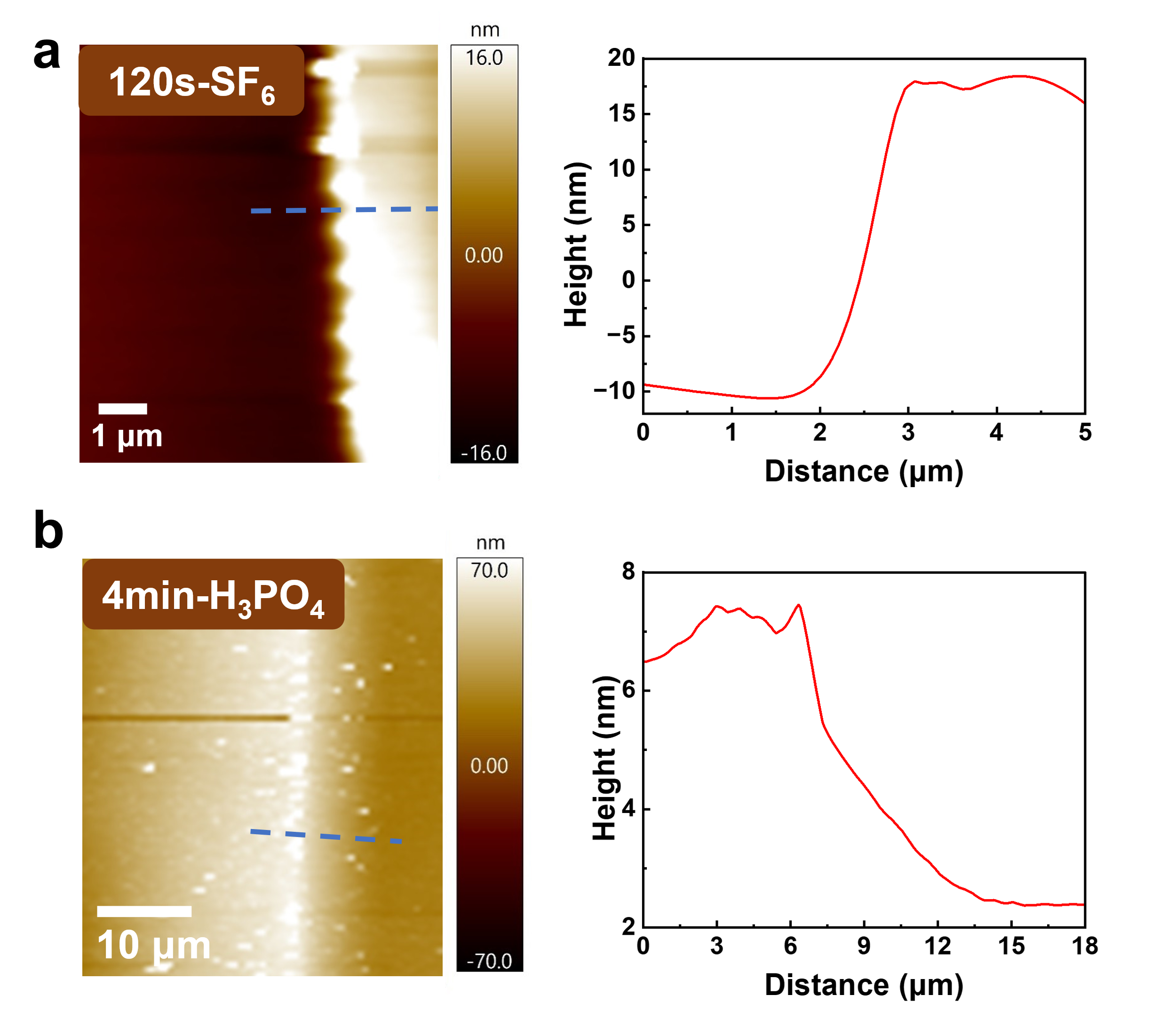


**Figure S4.** (a) The AFM morphology and height difference of Al_2_O_3_ after the 120 s dry etch of SF_6_. (b) The AFM morphology and height difference of Al_2_O_3_ after the 4 min wet etch of H_3_PO_4_.


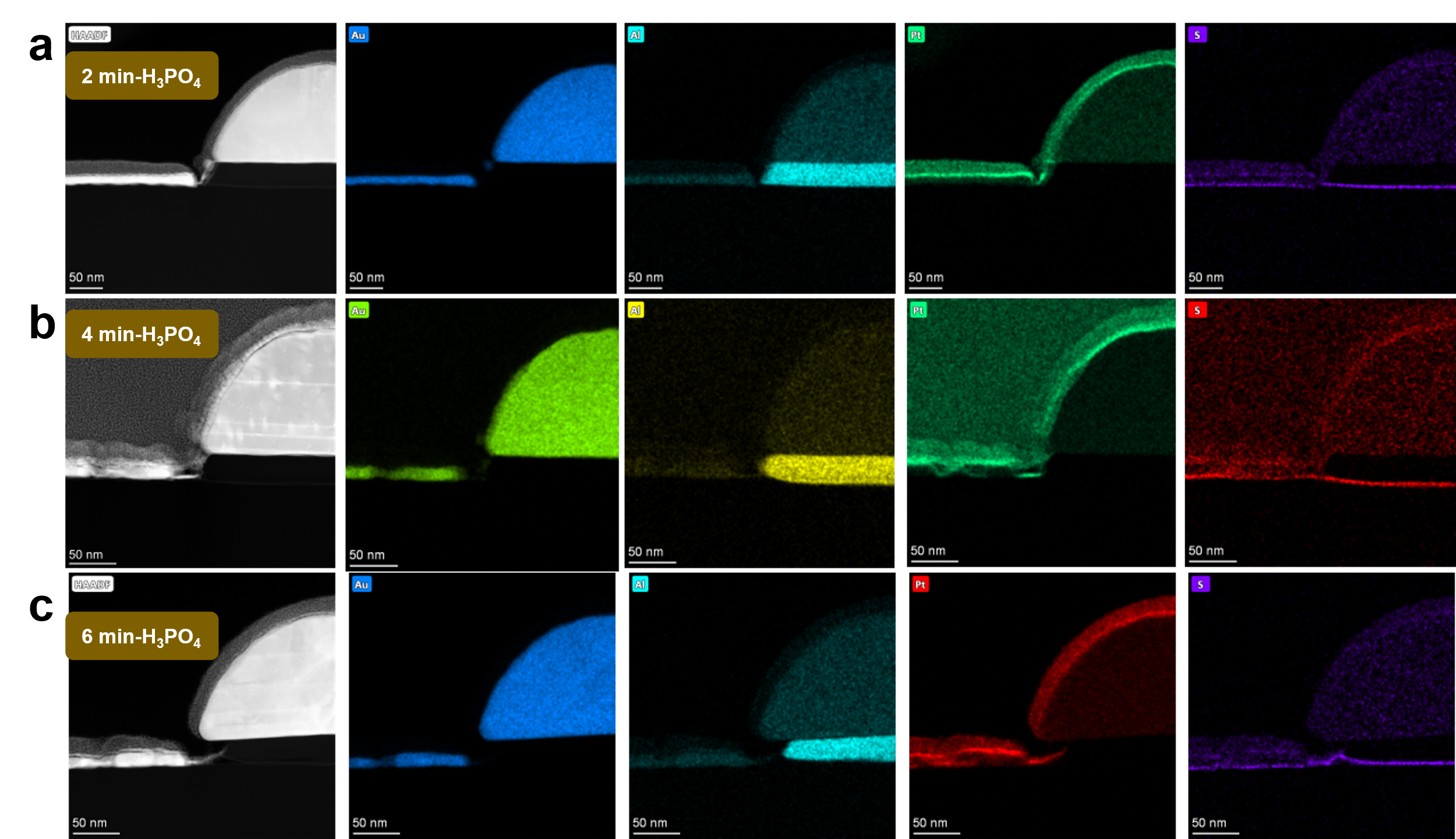


**Figure S5.** The HRTEM and EDS of the devices with (a) 2 min, (b) 4 min and (c) 6 min wet etch of H_3_PO_4_.


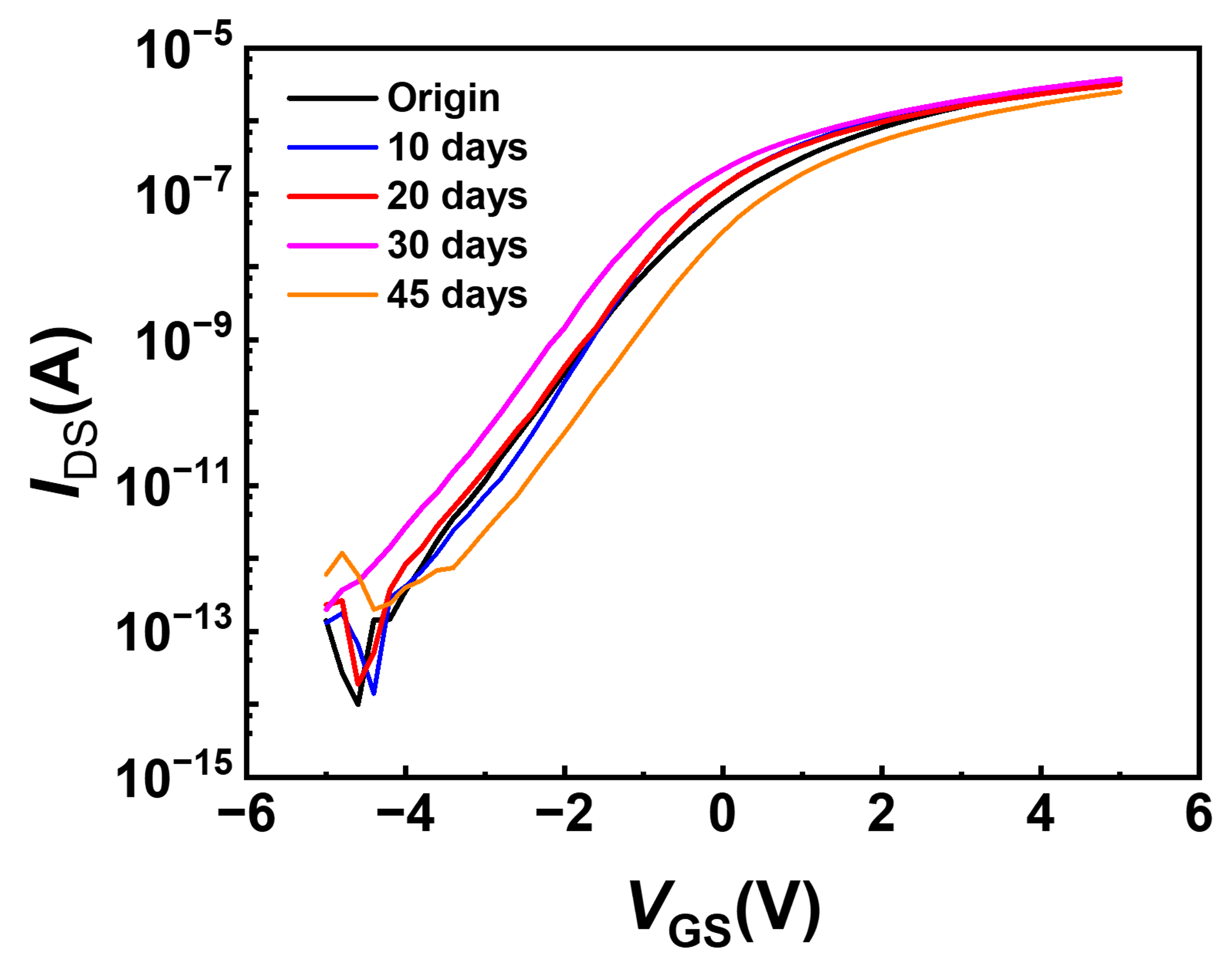


**Figure S6.** The transfer curves of the optimized device for origin, after 10 days, after 20 days, after 30days and after 45 days.


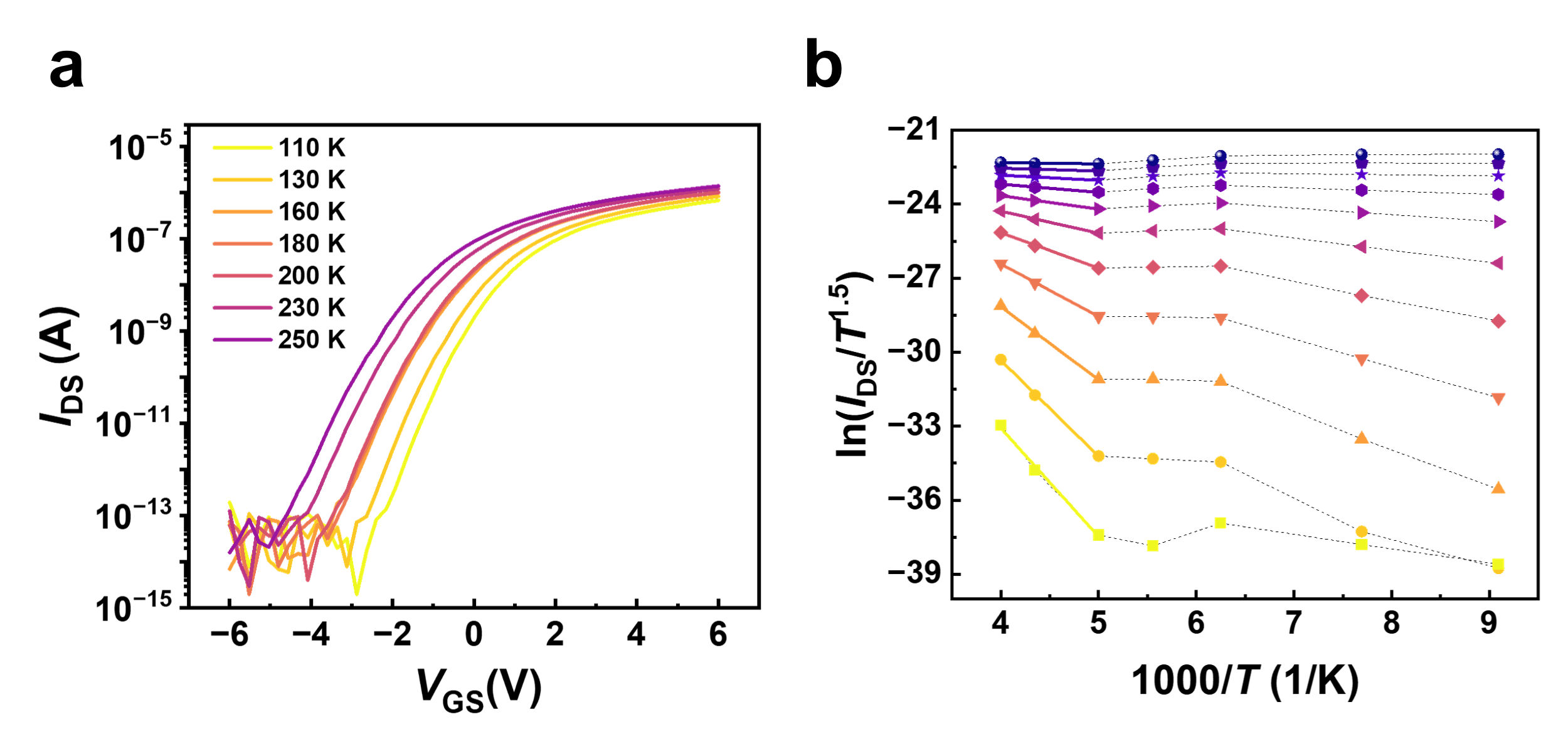


**Figure S7.** The I_DS_–V_GS_ characteristic curves (a) and the Arrhenius plots(b) of the self-aligned MoS_2_ FET at various temperatures from 110K to 250K.

**The calculation of Schottky barriers:** We extracted the Schottky barriers by using the Arrhenius equation, the drain current (I_DS_) thermally injected from the metal-TMD contact into the TMD channel through a reverse-biased Schottky barrier can be expressed as:

$I_{DS}=A_{2D}^{*}T^{1.5}\exp\left( -\frac{\Phi_{b}}{k_{B}T} \right)[1-\exp\left( -\frac{V_{DS}}{k_{B}T} \right)]$ (1)

Where $A_{2D}^{*}=q\frac{{(8\Pi{k_{B}}^{3}m^{*})}^{\frac{1}{2}}}{h^{2}}$ is the Richardson constant for a 2D system, T is the temperature, kB is Boltzmann’s constant, q is elementary charge, and Φ_b_ is the effective contact barrier height at a given gate-source voltage (V_GS_). If V_DS_ ≫ k_B_T, equation (1) is simplified to:

$$I_{DS}\approx A_{2D}^{*}T^{1.5}\exp\left( -\frac{\Phi_{b}}{k_{B}T} \right)$$

$$\ln\left( \frac{I_{DS}}{T^{1.5}} \right)\approx-\frac{\Phi_{b}}{k_{B}T}+C$$

The Φ_b_ obtained directly reflects the overall electrical behavior of the device. Thermionic emission is the dominant process when the gate voltage is lower than V_FB_. Therefore, accurate Φ_SBH_ can be extracted under flat-band voltage conditions (V_BG_ = V_FB_) in Figure 2e.


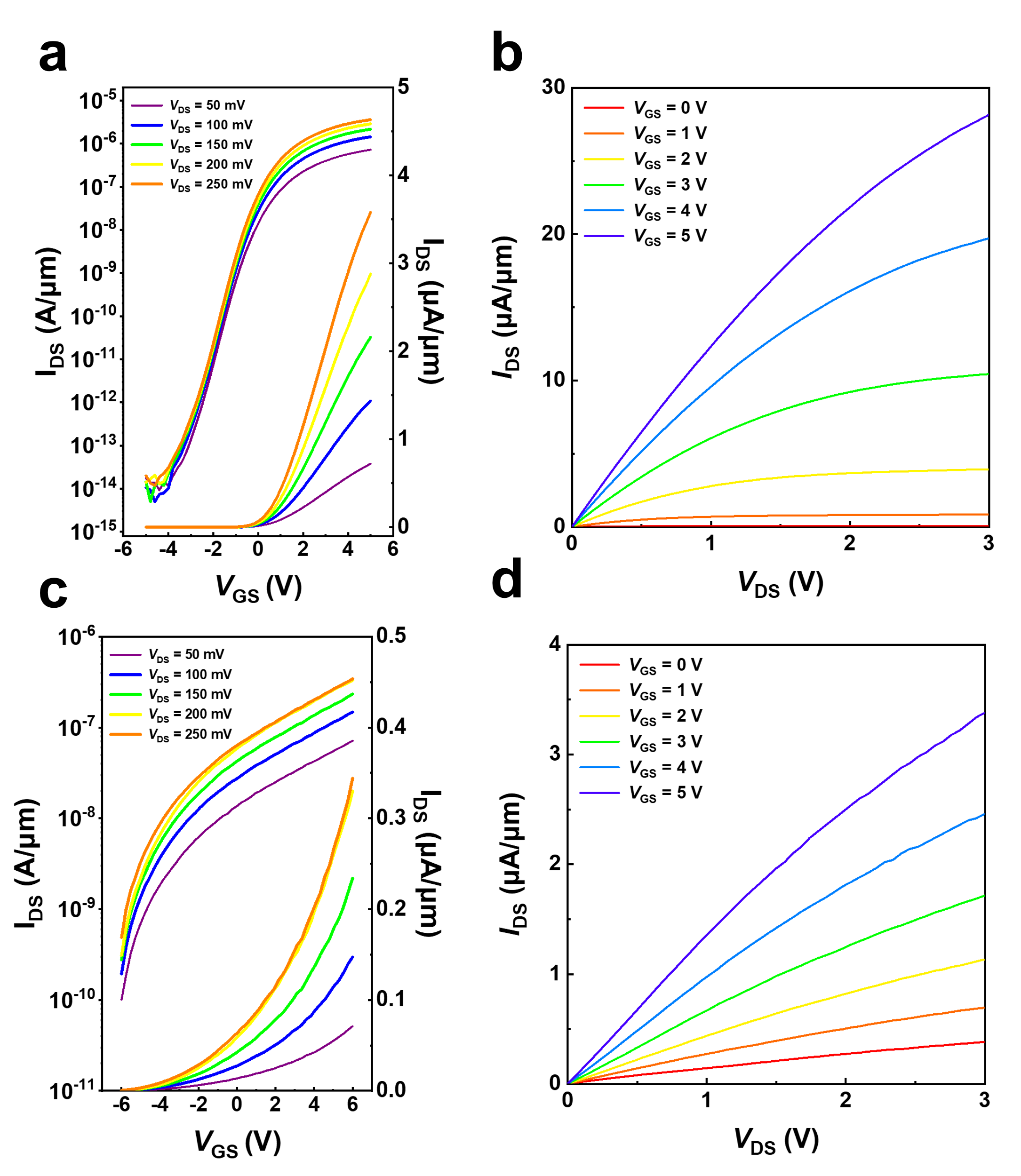


**Figure S8.** The transfer curves at *V*_DS_ from 50 mV to 250 mV and output curves at *V*_GS_ from 0 V to 5 V of the optimized device (a, b) and as-fabricated device (c, d)


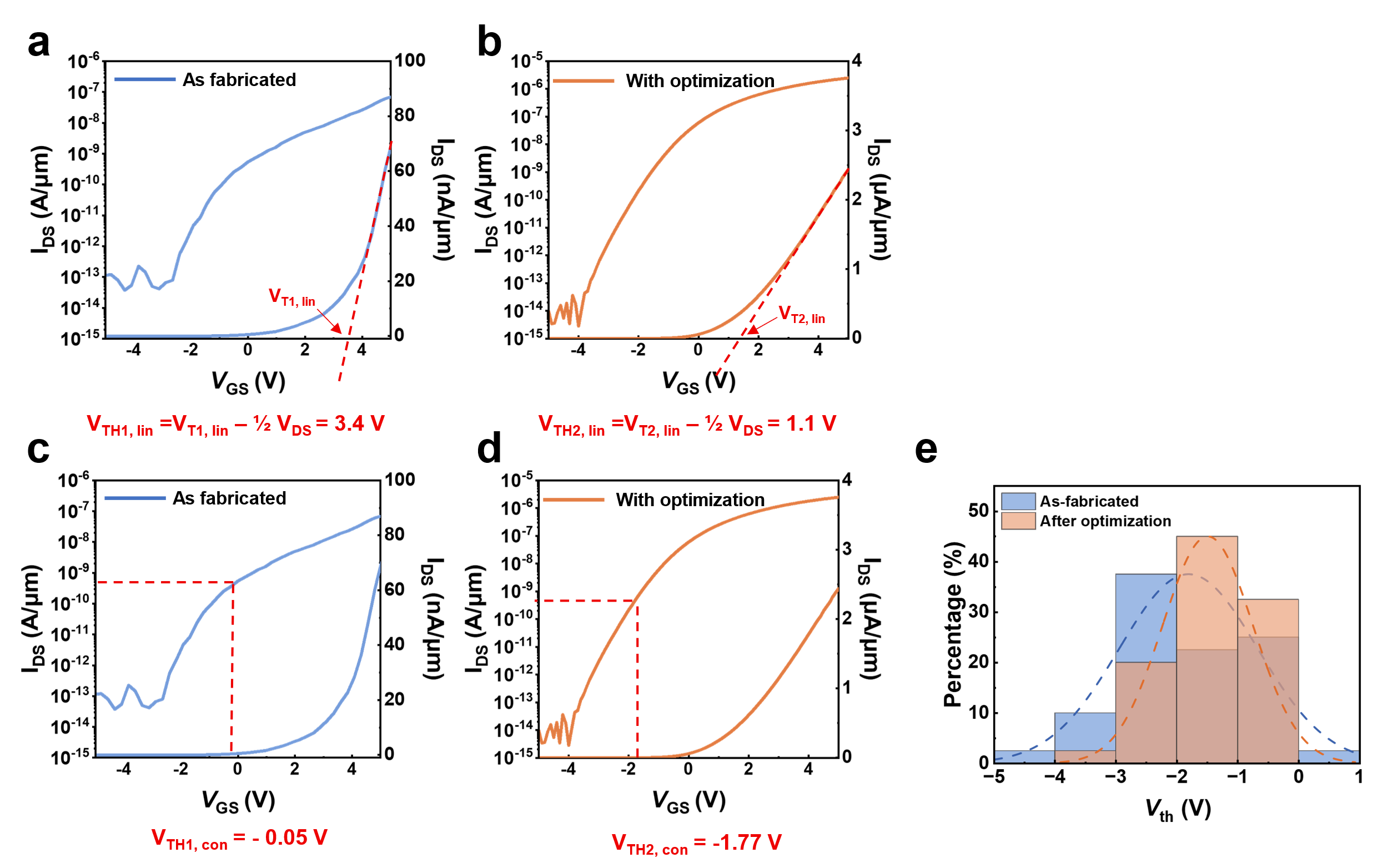


**Figure S9**. *V*_th_ extraction using linear extrapolation and constant current methods. (a), (b) Transfer characteristics of optimized and as-fabricated devices, with *V*_th_ extracted using the linear extrapolation method (1.1 V and 3.4 V, respectively). (c), (d) Transfer characteristics of optimized and as-fabricated devices, with *V*_th_ extracted measured using the constant current method at 100 pA/μm*W/L (-1.77 V and -0.05 V, respectively). (e) Statistical distribution of *V*_th_ of the as-fabricated and optimized self-aligned MoS_2_ TG-FETs.


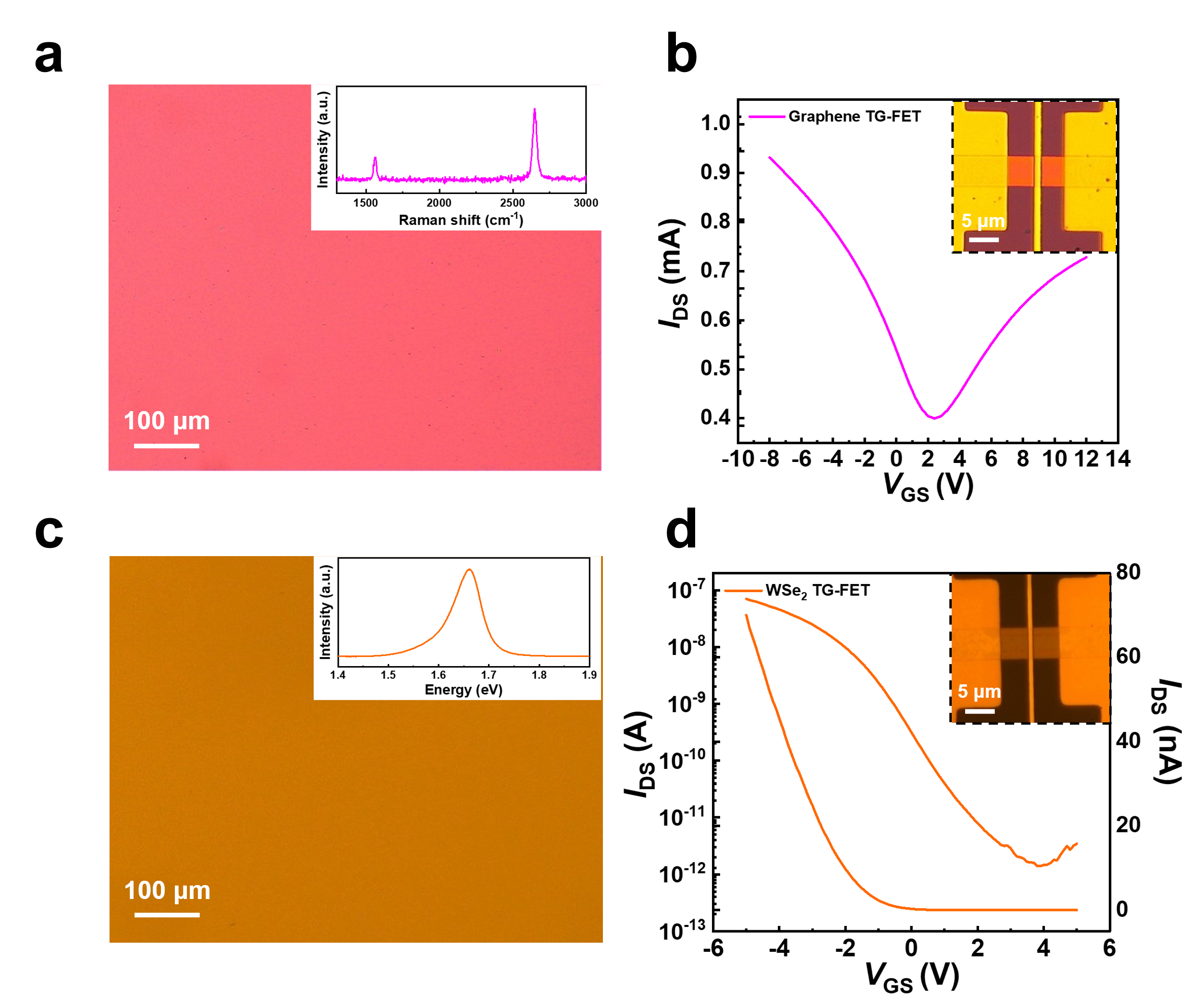


**Figure S10**. Self-aligned top-gate devices based on other 2D materials (graphene and WSe_2_). (a) Optical micrograph of the monolayer graphene, with the corresponding Raman spectra shown in the inset. (b) Transfer characteristics of the self-aligned top-gate device based on monolayer graphene (*V*_DS_ = 1 V), with the optical micrograph of the device shown in the inset. (c) Optical micrograph and the PL spectra (inset) of the monolayer WSe_2_. (d) Transfer characteristics (*V*_DS_ = 1 V) and the optical micrograph (inset) of the self-aligned top-gate device based on monolayer WSe_2_.
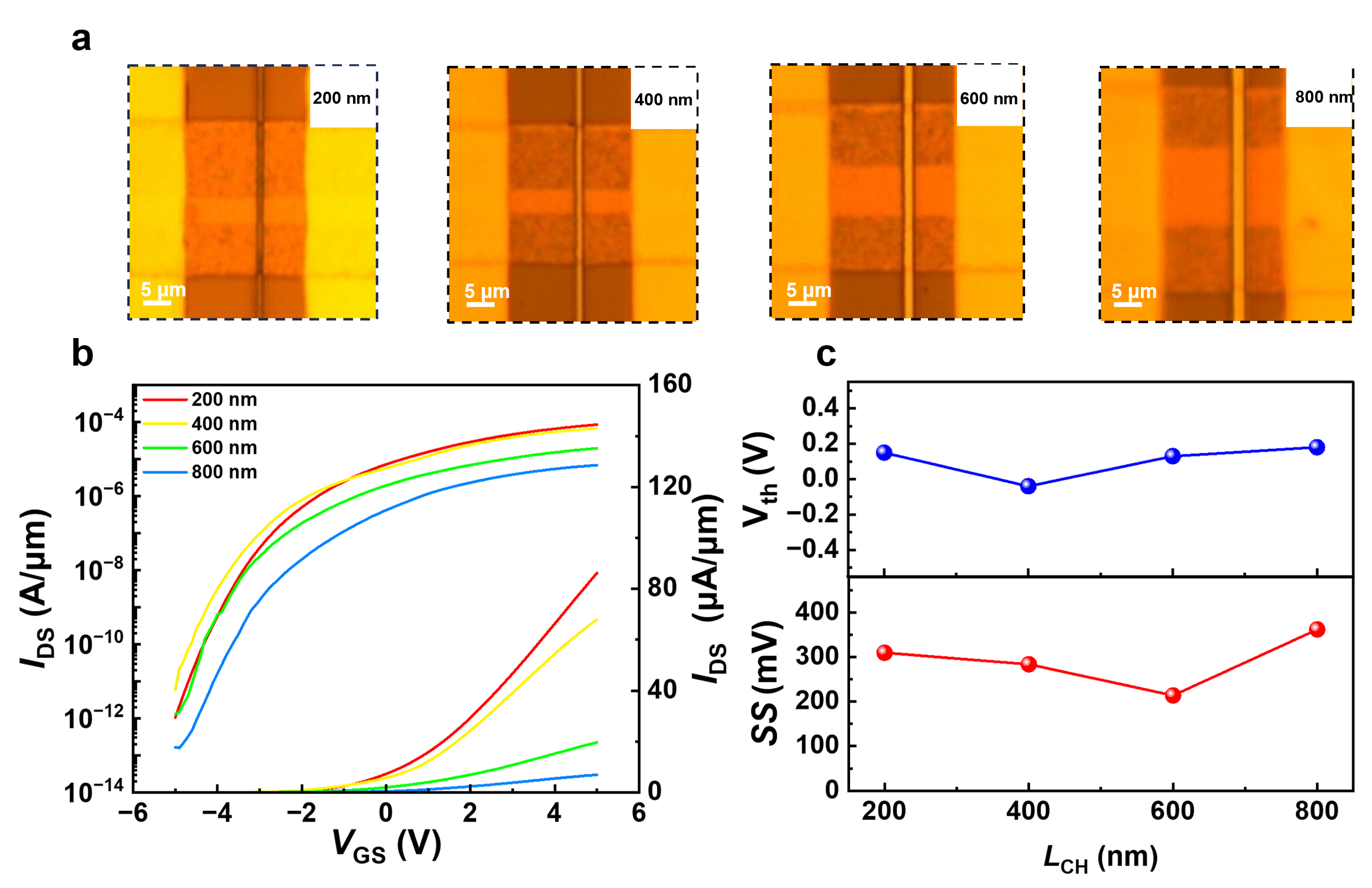


**Figure S11.** Optical images (a), transfer curves (b), *V*_th_ and *SS* (c), of self-aligned MoS₂ FETs with channel lengths ranging from 800 nm to 200 nm.


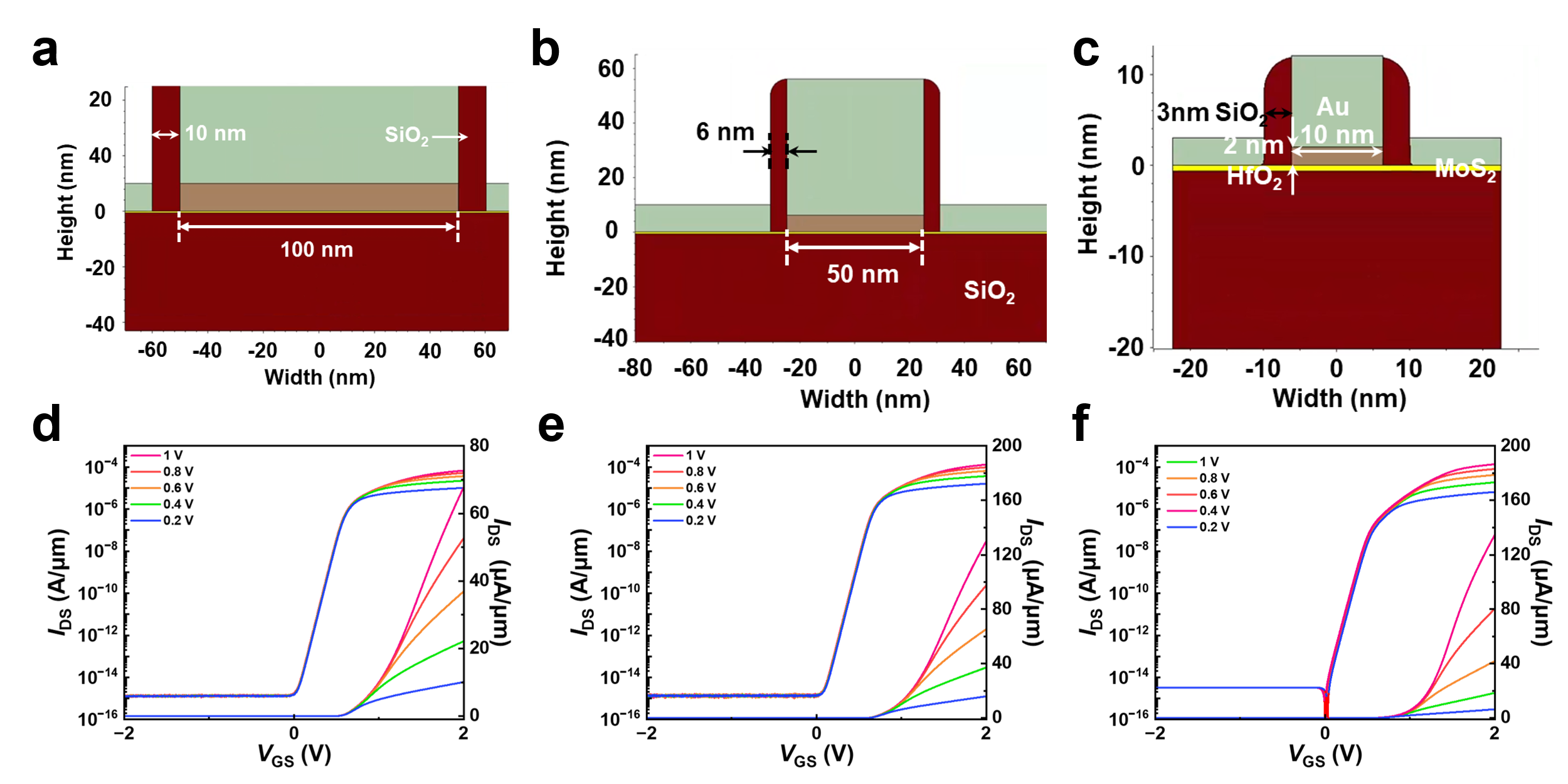


**Figure S12**. TCAD simulation diagrams of self-aligned TG-FET with 100 nm channel length and 10 nm sidewall width (a), 50 nm channel length and 6 nm sidewall width (b), 10 nm channel length and 3 nm sidewall width (c). TCAD simulated transfer curves of self-aligned TG-FET with 100 nm channel length and 10 nm sidewall width (d), 50 nm channel length and 6 nm sidewall width (e), and 10 nm channel length and 3 nm sidewall width (f).


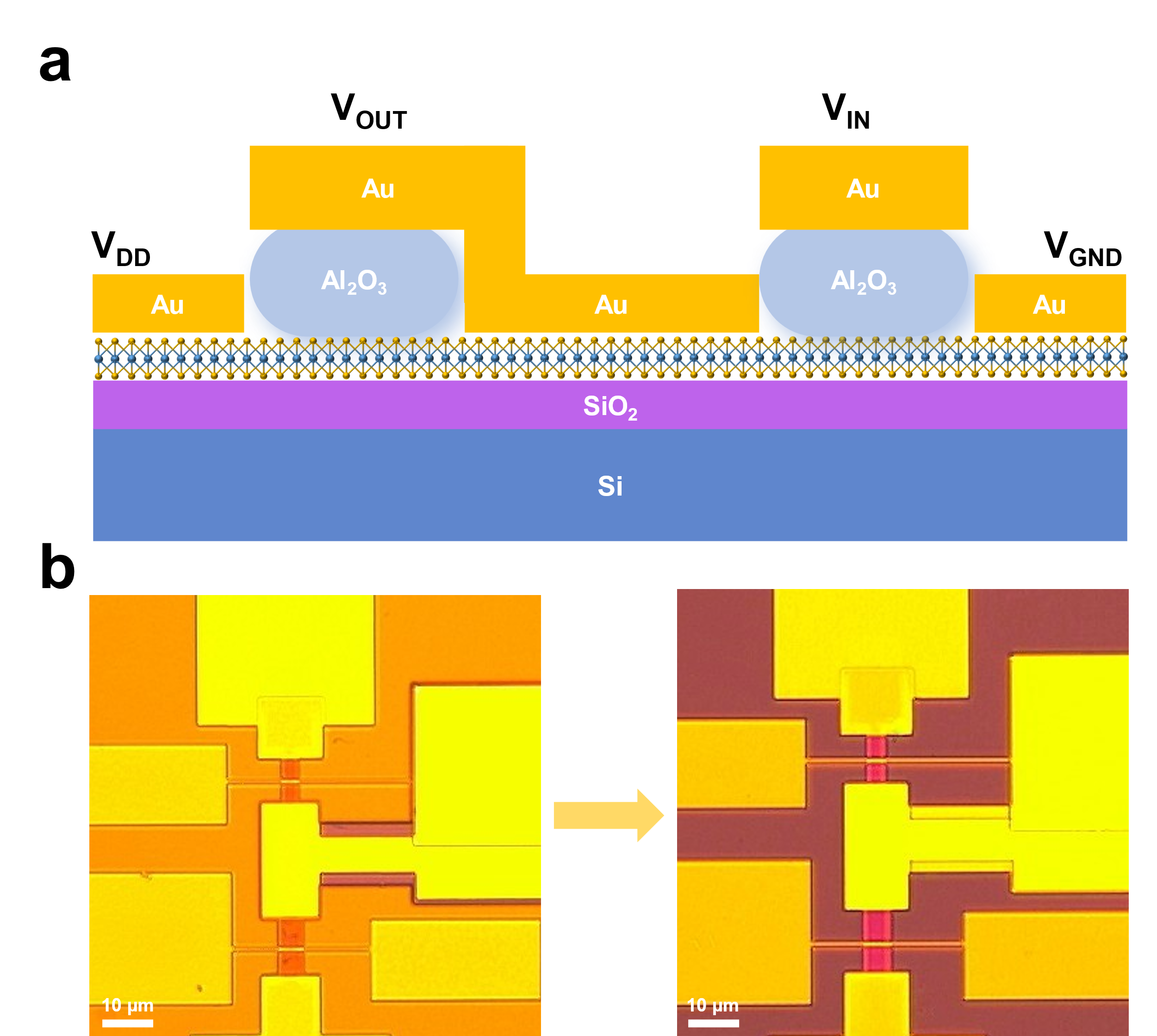


**Figure S13.** (a) Schematic of the inverters fabricated by the self-aligned MoS_2_ top-gate devices. (b) Optical images of the additional processes of the inverters.


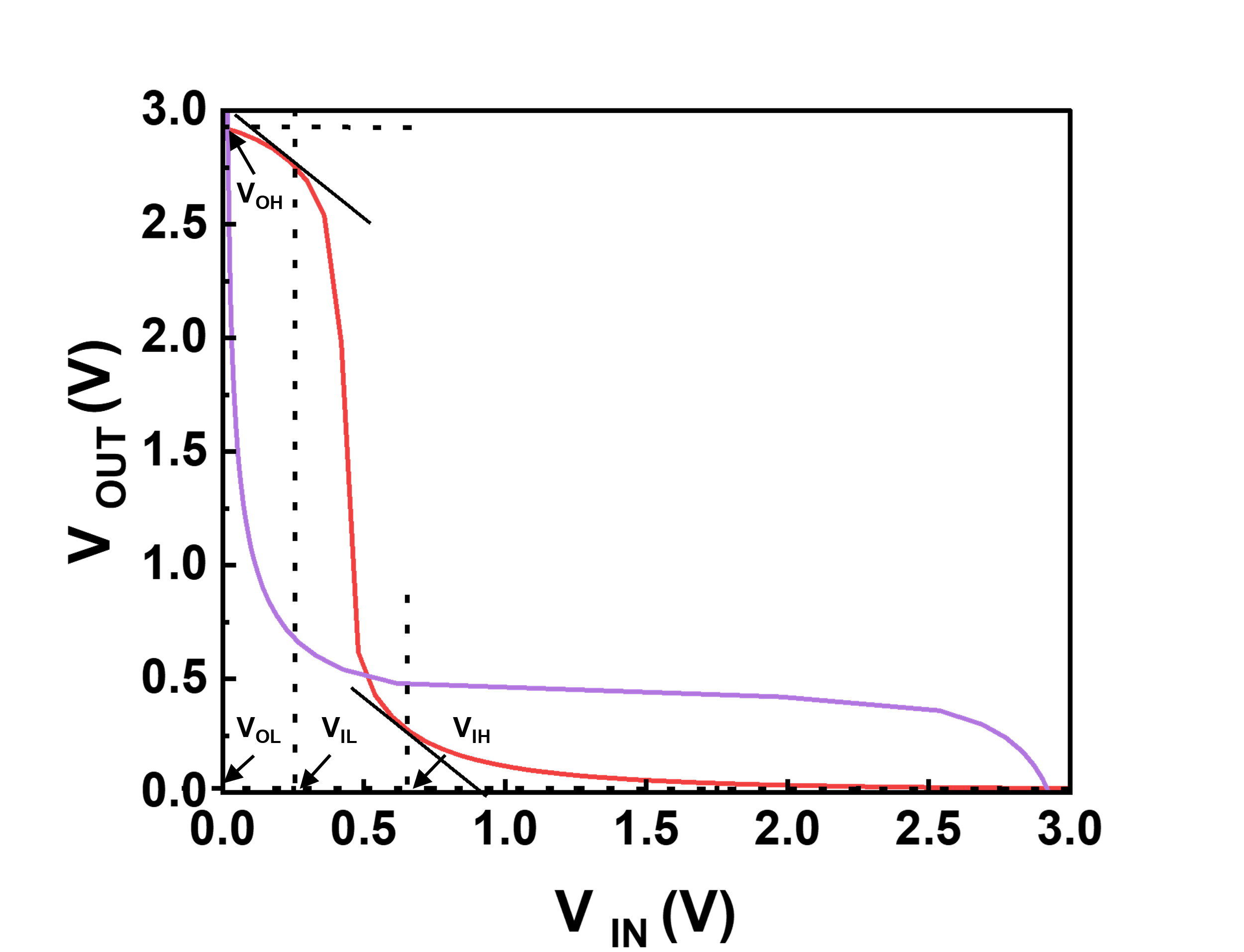


**Figure S14.** The voltage transfer characteristics (red line) and the mirror reflection (purple line) of the self-aligned inverters at V_DD_=3V. The V_OH_, V_OL_, V_IL_, and V_IH_ represent the minimum high output voltage, maximum low output voltage, maximum low input voltage, and minimum high input voltage for the inverter, respectively.


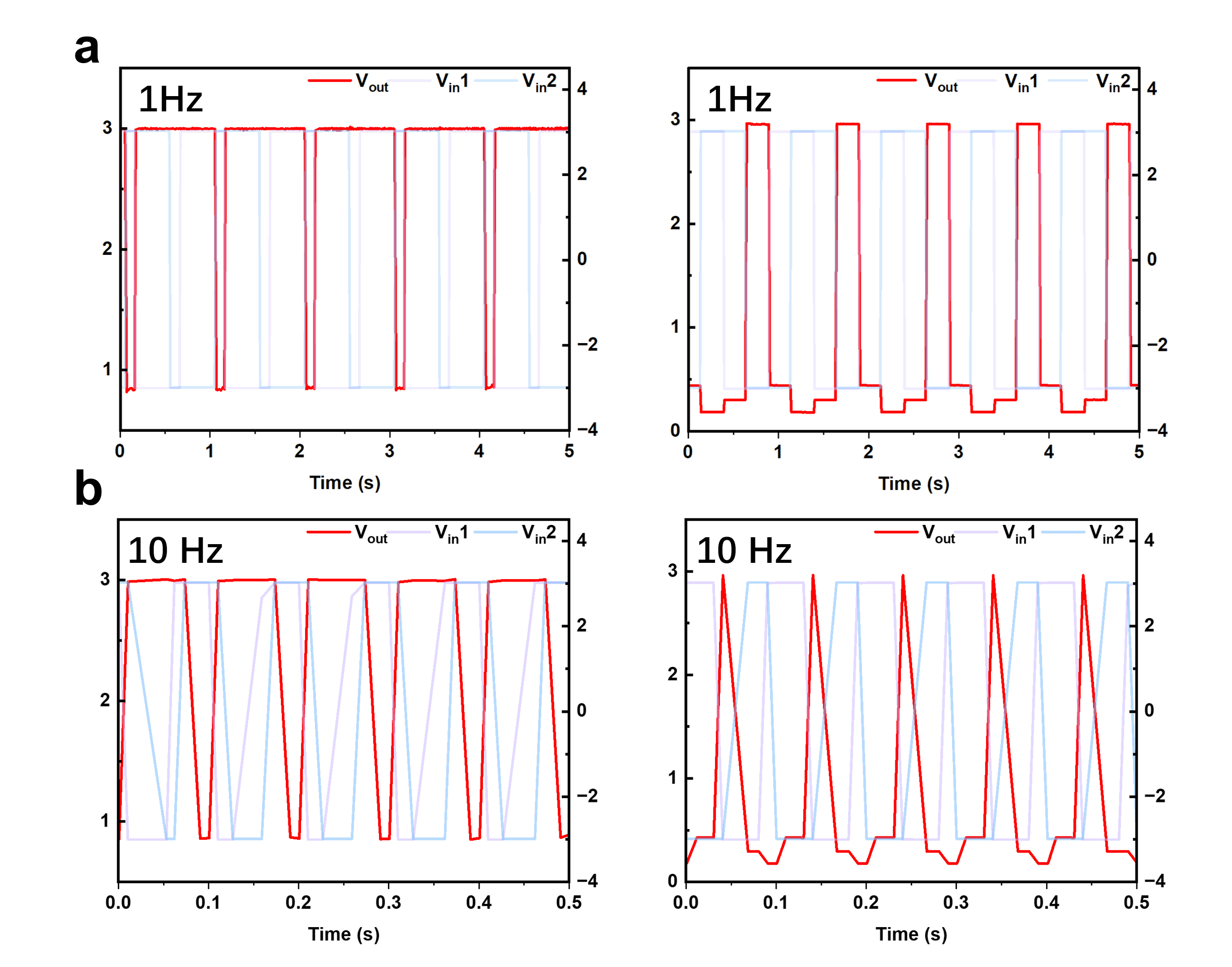


**Figure S15.** The input-output logic functions of the NAND and NOR at 1 Hz (a) and 10 Hz (b).


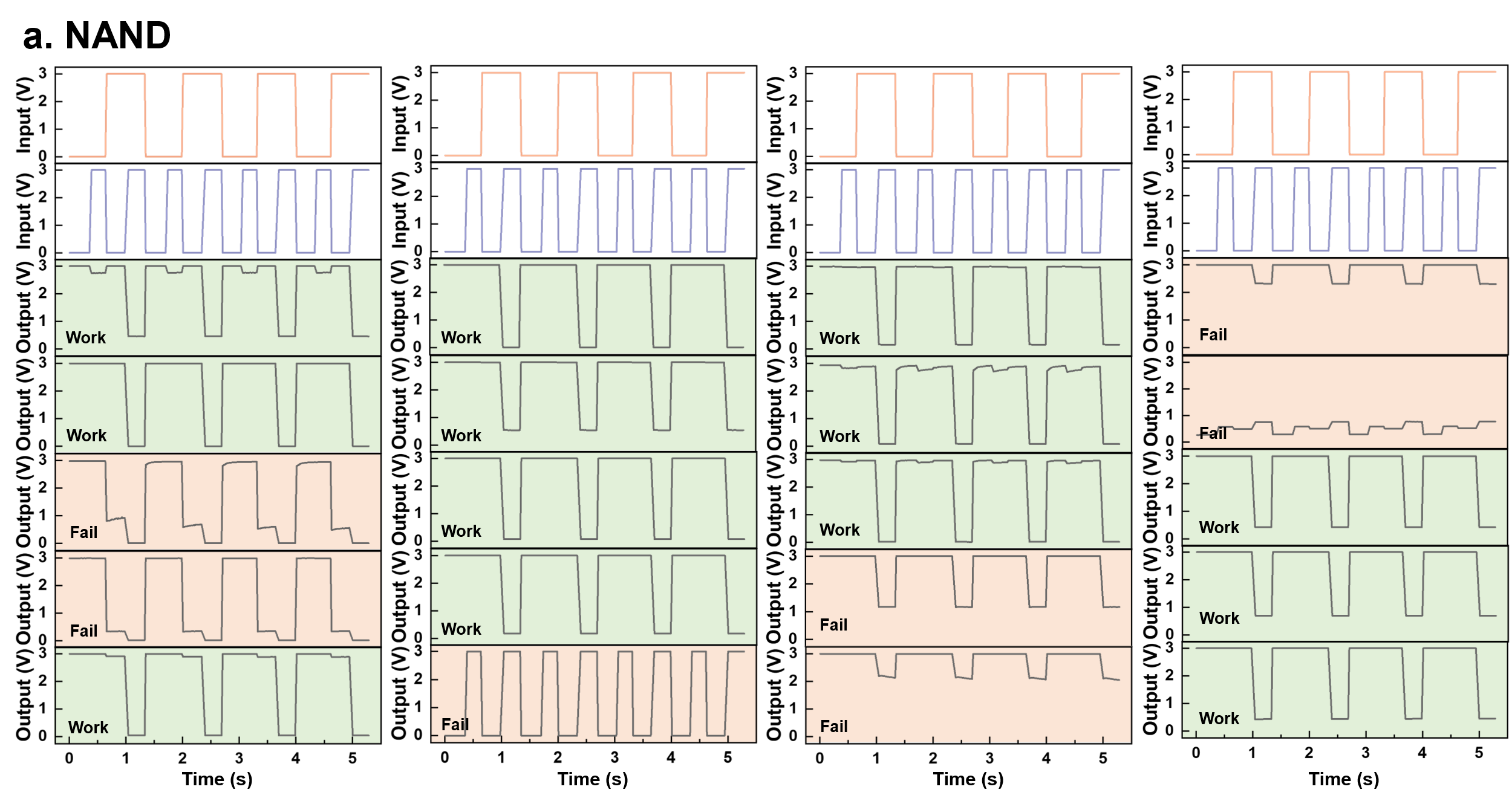


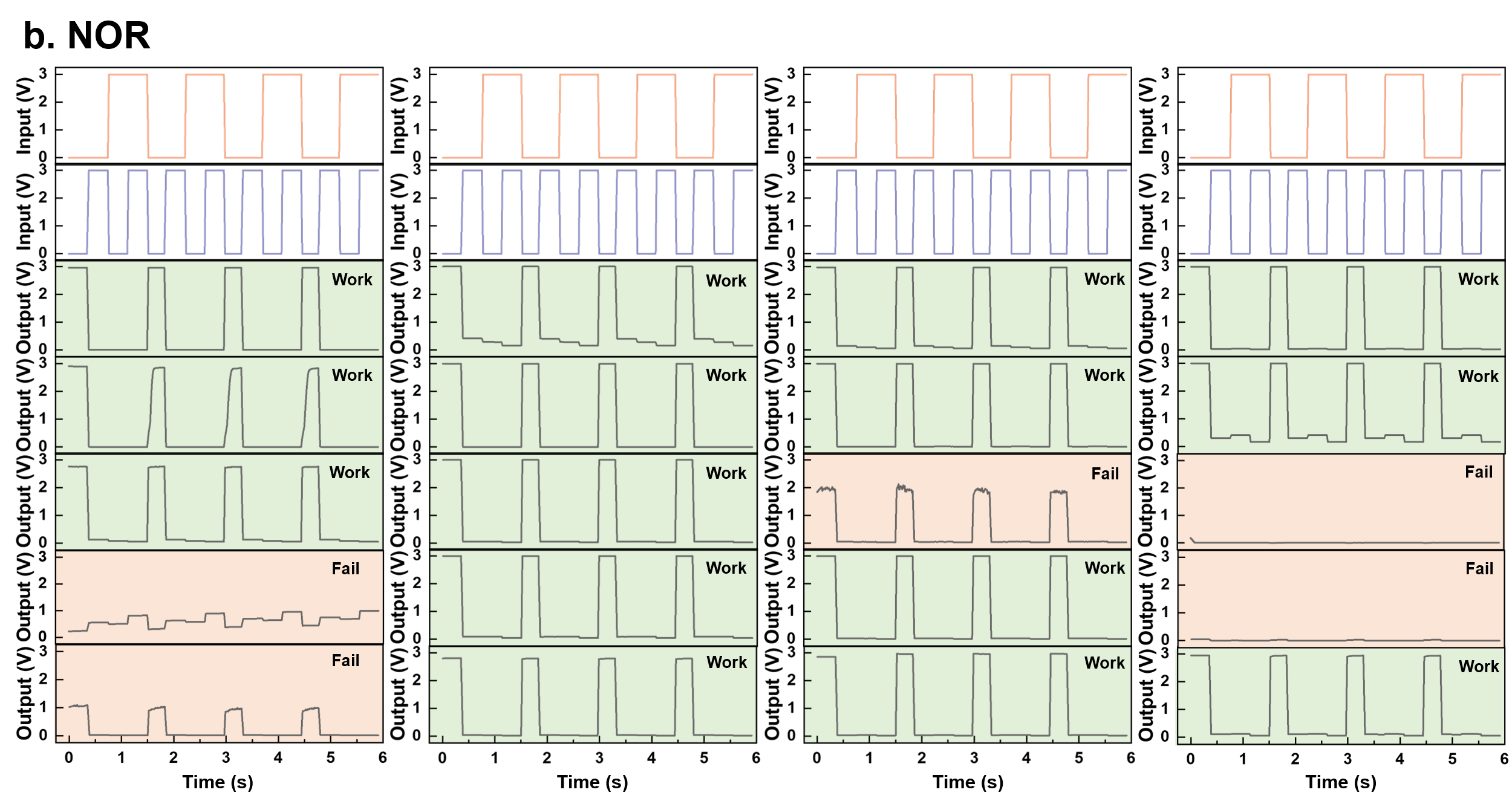
**Figure S16**. The statistics analysis of the NAND logic units (a) and NOR logic units (b).
